# Exploring the Potential of Al(III) Photosensitizers for Energy Transfer Reactions

**DOI:** 10.1021/acs.inorgchem.4c01922

**Published:** 2024-08-12

**Authors:** Volkan Caliskanyürek, Anastasiia Riabchunova, Stephan Kupfer, Fan Ma, Jia-Wei Wang, Michael Karnahl

**Affiliations:** † Department of Energy Conversion, Institute of Physical and Theoretical Chemistry, Technische Universität Braunschweig, Rebenring 31, 38106 Braunschweig, Germany; ‡ Institute of Physical Chemistry, Friedrich Schiller University Jena, 07743 Jena, Germany; § School of Chemical Engineering and Technology, Sun Yat-sen University, Zhuhai 519082, China

## Abstract

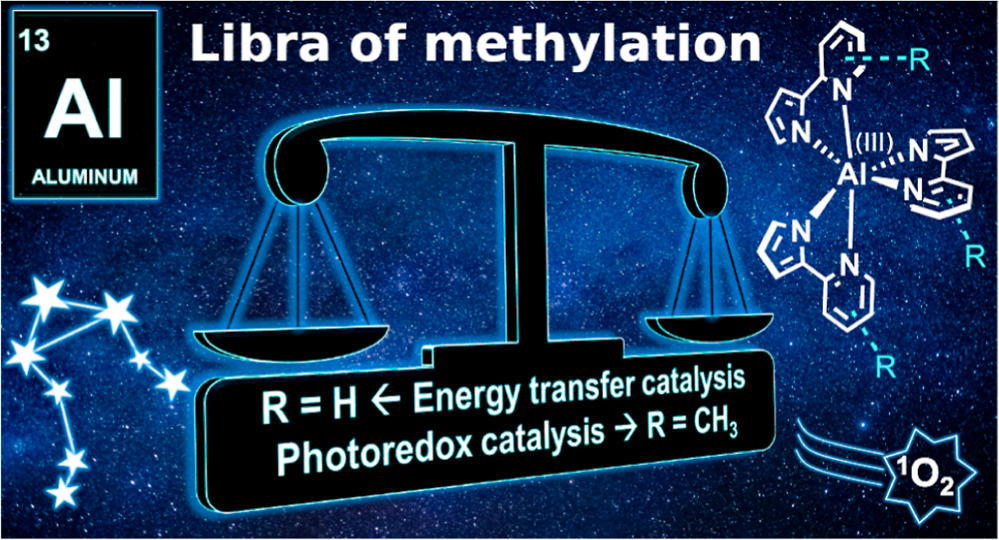

Three homoleptic Al(III) complexes (**Al1**–**Al3**) with different degrees of methylation at the 2-pyridylpyrrolide ligand were systematically tested for their function as photosensitizers (PS) in two types of energy transfer reactions. First, in the generation of reactive singlet oxygen (^1^O_2_), and second, in the isomerization of (*E*)- to (*Z*)-stilbene. ^1^O_2_ was directly evidenced by its characteristic NIR emission at around 1276 nm and indirectly by the reaction with an organic substrate [e.g. 2,5-diphenylfuran (DPF)] using *in situ* UV/vis spectroscopy. In a previous study, the presence of additional methyl groups was found to be beneficial for the photocatalytic reduction of CO_2_ to CO, but here **Al1** without any methyl groups exhibits superior performance. To rationalize this behavior, a combination of photophysical experiments (absorption, emission and excited state lifetimes) together with photostability measurements and scalar-relativistic time-dependent density functional theory calculations was applied. As a result, **Al1** exhibited the highest emission quantum yield (64%), the longest emission lifetime (8.7 ns) and the best photostability under the reaction conditions required for the energy transfer reactions (e.g. in aerated chloroform). Moreover, **Al1** provided the highest rate constant (0.043 min^–1^) for the photocatalytic oxygenation of DPF, outperforming even noble metal-based competitors such as [Ru(bpy)_3_]^2+^. Finally, its superior photostability enabled a long-term test (7 h), in which **Al1** was successfully recycled seven times, underlining the high potential of this new class of earth-abundant PSs.

## Abstract 2

This study deals with three Al(III) based photosensitizers (PS) that possess different degrees of ligand methylation. While these PS have been studied in photoredox catalysis (CO_2_ reduction), other catalytic applications such as energy transfer reactions have not yet been investigated. Therefore, the potential of these Al(III) PSs in two different energy transfer reactions, namely the photooxidation of 2,5-diphenylfuran by the generation of singlet oxygen and the isomerization of *E*- to Z-stilbene, was elucidated.

## Introduction

Solar energy has an enormous potential as a clean and abundant source of energy, but it needs to be captured and converted into storable forms.^1−4^ One method is to use photochemical reactions to produce solar fuels, which are energy-rich compounds that can be stored, transported, and converted back to their original state when needed.^5−7^ In addition, the application of visible light to drive organic transformations has attracted much attention in recent years.^8−10^ There, light is used as a reagent and energy source to enable a large variety of reactions, such as the isomerization of alkenes,^11−13^ various photocycloaddition reactions,^8,14^ reductive dehalogenations,^15,16^ α-functionalization of amines^17,18^ or the generation of reactive singlet oxygen (^1^O_2_).^19−21^ As a result, photoredox catalysis provides access to the selective production of a wide range of fine chemicals and pharmaceuticals under mild conditions.^8,14,22^ All these photocatalytic reactions require the design and application of efficient, inexpensive and sustainable photosensitizers (PS) or photoredox catalysts for light harvesting and primary charge separation as a common feature.^23−26^

**Figure 1:**
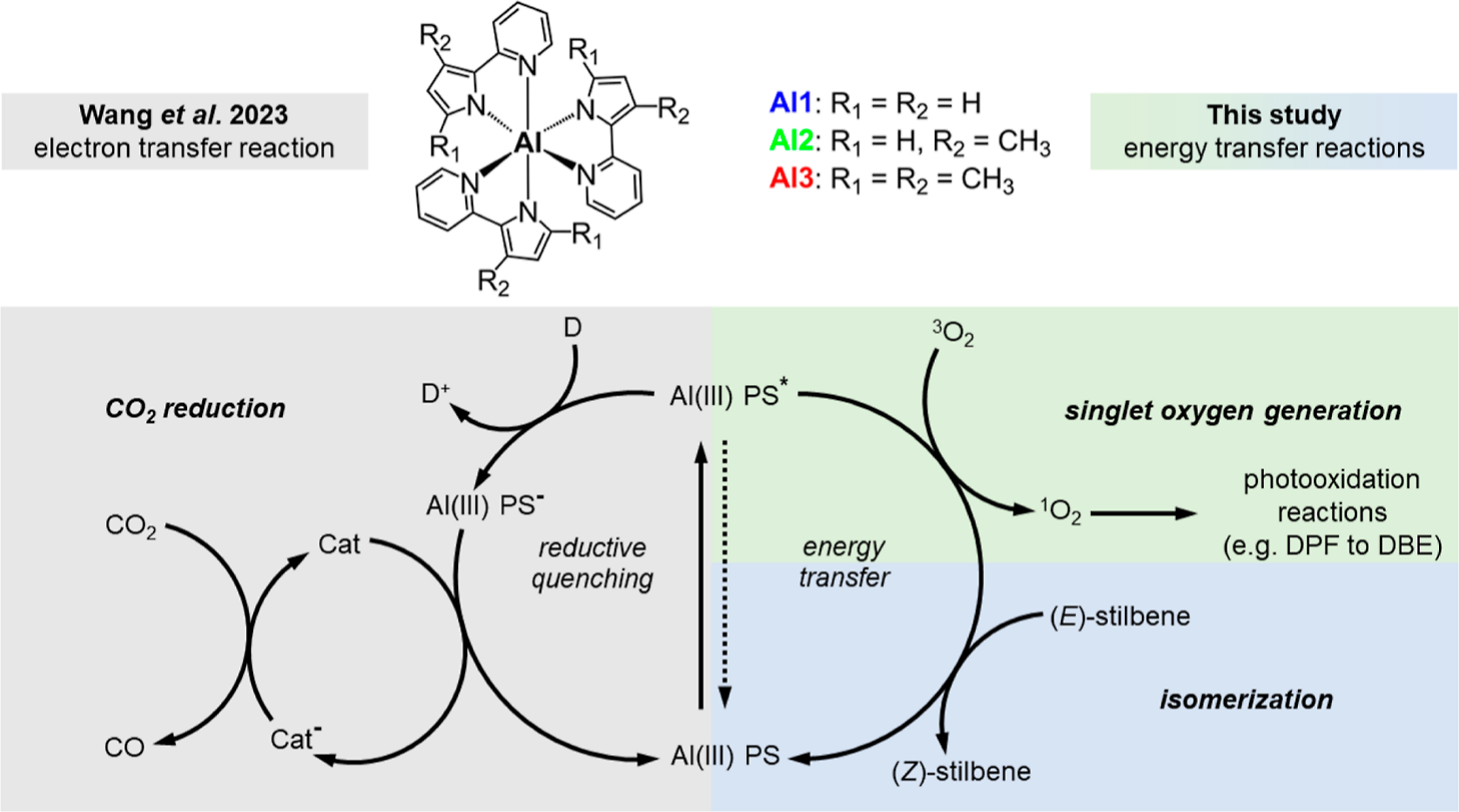
Molecular structures of **Al1–3** and general mechanistic pathways of photoredox catalysis. The excited aluminum photosensitizer Al(III) PS* can be reductively quenched by a sacrificial electron donor D to yield Al(III) PS^–^ (left side). The reduced Al(III) PS^–^ can then transfer an electron to a separate catalyst for the reduction of CO_2_ to CO (previous study).^1^ Alternatively, Al(III) PS* can directly transfer energy (right side) to ^3^O_2_ to generate ^1^O_2_ or to an organic substrate such as (*E*)-stilbene (this study).

Upon light-irradiation, the ground state PS is excited to form PS* (Figure 1).^14,24,27^ Subsequently, PS* can then either initiate a one-electron oxidation of a donor molecule (reductive quenching), resulting in the formation of a reduced PS^–^, or it can cause an one-electron reduction of an acceptor (oxidative quenching), yielding PS^+^.^14,24,27^ A major advantage of such an intermolecular system is its great flexibility and the ability to tune the PS and catalyst independently of each other.^27−29^

Alternatively, PS* can directly transfer energy to a suitable organic substrate (Figure 1—right side) without changing the redox state of the PS.^14,23,24^ The resulting electronically excited substrate S* reacts quite differently than in its ground state, opening up some unique possibilities in the field of organic synthesis. Prominent examples for such energy transfer catalysis are the isomerization of alkenes or the generation of ^1^O_2_ (Figure 1).^30−32^ In this case, ^1^O_2_ is typically produced *via* energy transfer from the triplet excited state of a suitable PS to triplet oxygen (^3^O_2_).^31−33^ Only after intersystem crossing (ISC) from a singlet to a triplet excited state, the ^3^PS* can then transfer its energy to ^3^O_2_ to form the metastable ^1^O_2_. This highly energetic and reactive species of molecular O_2_ is of great interest for several applications, including various photooxidation reactions,^32^ the production of organic peroxides and in photodynamic therapy (PDT).^34−36^ In PDT light reacts with a PS (also called photodynamic drug), which generates reactive oxygen species (e.g. ^1^O_2_) to shrink or kill a tumor due to its cytotoxic properties.^34,37^

In general, there are three classes of PSs: transition metal-based complexes, organic dyes and semiconductors.^5,26,38^ However, the vast majority of light-driven reactions still rely on rare and precious metals, such as transition metal complexes based on Ru(II),^39−41^ Ir(III)^42−44^ and Re(I).^45,46^ Unfortunately, iridium is even rarer in the Earth‘s crust than gold and along with ruthenium and rhenium it is one of the scarcest and most expensive metals.^47,48^ Organic dyes are also frequently applied as a low-cost alternative, but generally suffer from lower photostability.^38,49^

Consequently, an increasing number of luminescent 3d transition metal complexes based on Cu(I),^50−54^ Cr(0/III),^55−59^ Fe(II/III)^47,60−62^ or Ni(0/II)^63,64^ have been reported as potential PSs in recent years.

In contrast, photoactive complexes using cheap and abundant main group metals such as Mg(II) or Al(III) are still largely unexplored, with only limited examples.^48,65−71^ In this context, our research team has successfully applied a systematic series of homoleptic Al(III) PSs featuring 2-pyridylpyrrolide ligands for the photocatalytic reduction of CO_2_ to CO in a recent study.^1^ In combination with an Fe(II)-quaterpyridine catalyst, this fully noble-metal-free system allowed for a selective (>99%) and highly efficient (maximum turnover number of 10,250) CO_2_ to CO conversion.^1^ Moreover, it was found that the stepwise introduction of additional methyl groups at the pyrrolide rings significantly improves the visible-light absorption, reducing power and photostability of the respective Al(III) complexes (Figure 1). This has made the multiply methylated complexes exceptionally good Al(III) based PSs.^1,68−70^ Transient absorption spectroscopy also revealed the existence of a nonemissive triplet excited state in addition to the luminescent (excited) singlet state.^1^

However, these Al(III) PSs have not yet been extensively tested in other photoredox catalytic reactions, and there has been no investigation of energy transfer reactions at all (Figure 1). Therefore, this study explores the potential of homoleptic Al(III) complexes for different energy transfer reactions in detail. On the one hand, the light-driven generation of reactive ^1^O_2_ and its subsequent application in oxygenation reactions was examined (Figure 1—right side). In this respect, the oxidation of 2,5-diphenylfuran (DPF) to *cis*-1,2-dibenzoylethylene (DBE) was chosen as a suitable reaction.^16,32,72^ On the other hand, the photocatalytic isomerization of (*E*)- to (*Z*)-stilbene was selected as second model reaction.^13,30,73^ In all photocatalytic measurements, light > 400 nm was used to prove that visible light is sufficient to enable the efficient use of Al(III) PSs.

In addition to the catalytic applications, structure–property relationships and in particular the effects of the different numbers of methyl substituents have been elucidated. Furthermore, the catalytic conditions have been optimized, as the production of ^1^O_2_, for example, is highly dependent on the solvent.^21,33,37^ To substantiate our results, relevant spectroscopic data were determined in additional solvents compared to the previous study.^1^ Scalar-relativistic time-dependent density functional theory (TDDFT) simulations [including spin–orbit coupling (SOC)] were performed to evaluate the nature of the electronic transitions upon light excitation and to identify possible subsequent relaxation pathways. Finally, the good photostability and resistance toward reactive ^1^O_2_ as well as the long-term use of these Al(III) PSs in recycling experiments were also demonstrated. The focus of this study is on the applicability of Al(III) PSs in various catalytic systems rather than providing a complete description of the underlying reaction mechanisms.

## Results and Discussion

### Singlet Oxygen Emission Measurements

Photochemically, ^1^O_2_ is typically generated by an energy transfer from the triplet excited state of a suitable PS.^19,31,33,74,75^ In molecular transition metal-based PS, there is a design tension between the creation of long-lived excited (triplet) states and the population of such states. Long-lived triplet states have low SOC, and therefore, their population, either by direct photoexcitation or by subsequent excited state relaxation is low.^76^ Increasing the SOC improves the efficiency of the triplet state population, but at the cost of decreasing the excited state lifetime.^76^ Indeed, Wang et al. provided transient absorption measurements, confirming a dark, but long-lived triplet state for **Al1–3** [2.13–1.44 μs in deaerated acetonitrile (CH_3_CN)].^1^ Therefore, this study is the first to investigate whether this previously characterized triplet state is suitable for the production of ^1^O_2_.

Singlet oxygen has a characteristic emission (phosphor-escence) in the near-infrared at around 1276 nm, which corresponds to the energy difference of ≈94 kJ·mol^–1^ between ^1^Δ_*g*_ (lowest singlet state) and ^3^Σ_*g*_^–^ (triplet ground state).^32,33,75,77^ Hence, **Al1–3** were measured in different solvents, including tetrahydrofuran (C_4_H_8_O), dichloro-methane (CH_2_Cl_2_) and chloroform (CHCl_3_), as the ^1^O_2_ emission strongly depends on the solvent (Figure S2).^33,78^ In the present case, CH_3_CN is not suitable due to the rapid deactivation of ^1^O_2_.^33,78,79^ Significant emission was only observed in CHCl_3_, albeit with low intensity, making it difficult to quantify the efficiencies of the three Al(III) PSs studied. This low intensity is due to the short lifetime of ^1^O_2_ in these solvents, with the shortest in C_4_H_8_O (20 μs) and the longest in CHCl_3_ (60 μs).^80,81^ To overcome this limitation, deuterated chloroform (CDCl_3_) can be used to increase the emission signal, because ^1^O_2_ is deactivated less rapidly in CDCl_3_ (7.0 ms).^33^ A dominant and competitive deactivation process is the direct decay of the excited singlet state of PS back to its ground state. Notably, deactivation within the singlet manifold is significantly hampered in CDCl_3_, which increases the probability of ISC, and thus, results in higher ^1^O_2_ emission intensities.^82^

**Figure 2:**
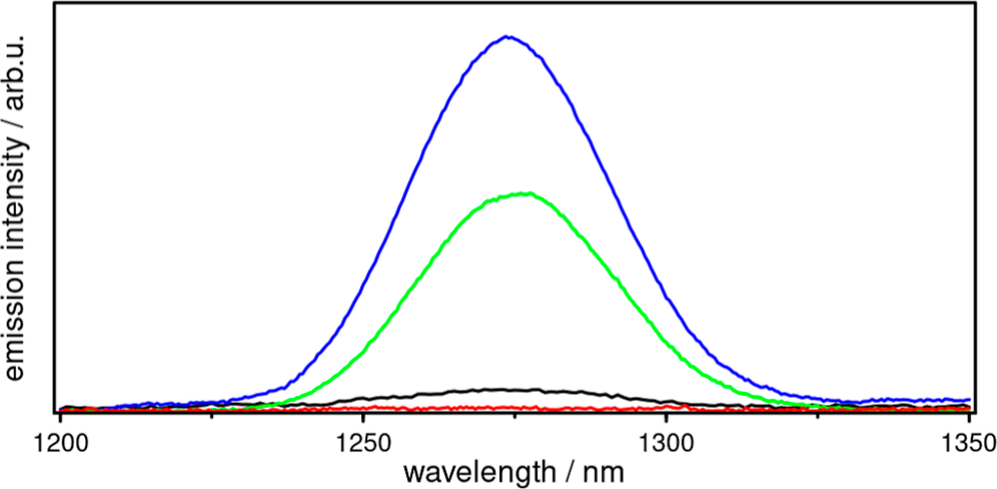
Singlet oxygen emission of **Al1** (blue, λ_exc_ = 372 nm), **Al2** (green, λ_exc_ = 385 nm), **Al3** (red, λ_exc_ = 400 nm) and **Alq**_**3**_ (black, λ_exc_ = 388 nm) in aerated CDCl_3_ with an optical density of 0.1 for λ_exc_.

Consequently, emission measurements in CDCl_3_ yield intense signals (Figure 2), showing that **Al1** and **Al2** generate ^1^O_2_, whereas **Al3** gives no evidence of ^1^O_2_ emission. At first glance, this seems to be in contrast to the results by Wang et al.,^1^ who reported superior photocatalytic CO_2_ to CO conversion using **Al3**. However, in the case of ^1^O_2_ production, the energy is transferred without changing the oxidation state of the PS, which corresponds to a completely different catalytic mechanism. Therefore, a higher degree of methylation improves the electron transfer properties, but seems to reduce the energy transfer capability of the Al(III) PS. To fully understand this reverse order of activity, the different stability toward ^1^O_2_ and subsequent oxidation reactions must also be considered, which is discussed in the following sections.

After the ^1^O_2_ emission measurements, 9,10-diphenyl-anthracene was added to the reaction mixture as a potent ^1^O_2_ scavenger.^83,84^ In consequence, the intense ^1^O_2_ signal was completely quenched (Figure S3), again proving that the observed NIR emission is indeed attributed to ^1^O_2_.^83−85^ The ^1^O_2_ quantum yield Φ(^1^O_2_) could not be determined in our case due to the lack of suitable references in CDCl_3_ (for further details see Section S5).

### Photophysical Properties in Chloroform

**Figure 3:**
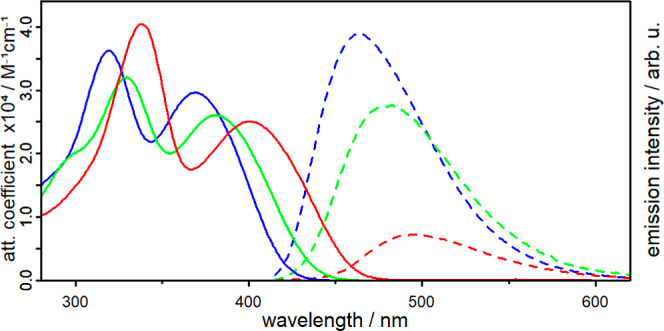
Molar attenuation coefficients (solid lines) and emission spectra (dotted lines) of **Al1** (blue), **Al2** (green) and **Al3** (red) in deaerated CHCl_3_. Emission measured with λ_exc_ = 405 nm and OD_405_ = 0.1.

To identify the structure–activity relationship for these Al PSs with respect to the observed ^1^O_2_ evolution, their photophysical properties were re-evaluated in CHCl_3_ solutions. The UV/vis absorption spectra reveal similar shapes for **Al1–3** in CHCl_3_ solution, each showing two broad bands (Figure 3). The absorbance is noticeably red-shifted from 369 nm (**Al1**) to 400 nm (**Al3**) (≈0.26 eV) with a higher degree of methylation (Table 1), which was also observed in CH_2_Cl_2_, C_4_H_8_O and CDCl_3_ (Figure S4) as well as in CH_3_CN.^1^ The nature of the electronic transitions was examined by TDDFT as well as scalar-relativistic TDDFT simulations (B3LYP XC functional and def2-SVP basis set).^86−89^ Furthermore, SOC values among the (excited) singlet and triplet states of interest were obtained to identify prominent pathways for ISC, i.e., population transfer from singlet excited states of **Al1**–**Al3** to the energetically close triplet states.^90,91^ Implicit solvent effects (CHCl_3_) were taken into account by means of a polarizable continuum model. Within the spin-free (pure singlet and triplet states) and the spin–orbit (linear combination of singlet and triplet states) picture, the calculated absorption spectra are in agreement with the experimental absorption spectra in CHCl_3_ (Figures S8 and S9). This also applies to the previously reported theoretical and experimental spectra in CH_3_CN.^1^

**Table 1:** Summary of the Photophysical Properties of **Al1–3** in Deaerated CHCl_3_^a^

Al(III) PS	λ_abs_/nm (ε/M^–1^ cm^–1^)	λ_em_/nm	Φ_em_/%	τ_em_/ns
**Al1**	320 (36,208), 369 (29,597)	467	63.7	8.7
**Al2**	325 (31,159), 379 (26,002)	483	49.1	8.6
**Al3**	338 (40,448), 400 (25,076)	500	13.0	7.2

a λ_exc_ = 369 nm (**Al1**), 379 nm (**Al2**) or 400 nm (**Al3**) with an OD_exc_ = 0.1.

**Figure 4:**
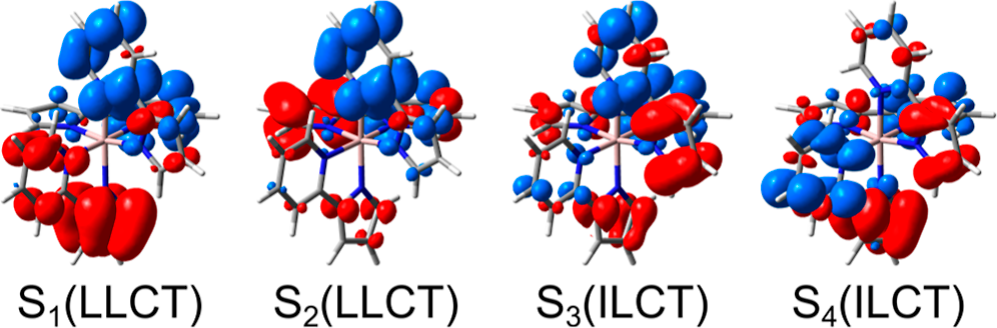
Electronic characters of prominent dipole-allowed transitions shown exemplarily for **Al1** as visualized by charge difference density plots of **Al1**. Electron density migrating from red to blue during excitation (densities are shown using an isovalue of 0.001).

In the following, the main focus is on the analysis of the theoretical results obtained on the basis of the dipole-allowed singlet–singlet transitions (spin-free). In the visible region, the quantum chemical calculations reveal ligand-to-ligand charge transfer (LLCT) transitions from the respective singlet ground state, i.e. S_1_ and S_2_, for all three Al(III) PS. In contrast, the absorbance in the UV region (<350 nm) is mainly related to several intraligand charge transfer (ILCT) transitions, e.g. into S_4_, S_9_, S_10_, S_11_, S_13_, S_14_ and S_20_ in the case of **Al1** (see S_1_–S_4_ in Figure 4). Notably, the inclusion of scalar-relativistic effects does not alter the results obtained by means of the spin-free states, as all relevant spin–orbit states are almost quantitatively governed by the respective spin free states. Hence, only very limited mixing between singlet and triplet states is predicted.^76,92−95^ This finding is well reflected by the weak spin–orbit couplings (SOC_max_ ≈ 1 cm^–1^) between the accessible and initially populated singlet excited states and the energetically close triplet states. In consequence, a rather slow and inefficient ISC is expected for all three PSs (Tables S5–S7), which is consistent with the previous results by Wang et al.^1^ This result is in agreement with the fact the investigated Al(III) complexes do not contain heavy elements which would typically exhibit greater spin orbit coupling contributions (e.g. Fe, Ru, Re, Ir or Pt).^76,92−95^ The similar shape of the UV/vis spectra and the comparable quantum chemical results imply that the electronic transitions are not significantly affected by the change in solvent. Details with respect to the underlying electronic transitions are collected in the Supporting Information (Section S4). The calculated absorption spectra further reveal a red shift of the LLCT region with a higher degree of methylation (Figure S8), which is also observed in the measured absorption spectra (Figure 3).

The emission spectra reveal a clearly red-shifted emission maximum λ_em,max_ (Δ = 1413 cm^–1^) from 467 nm (**Al1**) to 500 nm (**Al3**) with a higher degree of ligand methylation (Table 1). In addition, there are significant differences in the emission intensities and emission quantum yields Φ_em_. The highest emission intensity was observed for **Al1**, a medium one for **Al2** and the lowest for **Al3** (Figure 3). To determine the emission quantum yields, a commercially available Al(III)-based reference compound, namely aluminum-tris(8-hydroxychinolin) (**Alq**_**3**_, Figure S13), was used. **Alq**_**3**_ was selected due to its chemical similarity to **Al1–3** and matching excitation energies, as well as its well-documented quantum yield (Φ_em_ = 11.6% at λ_exc_ = 405 nm in CH_3_CN).^1,68,96^ The Φ_em_ of all Al(III) complexes in CHCl_3_ change significantly when compared to their values in CH_3_CN, as previously reported by Wang et al.^1,96^ The Φ_em_ increases from 49.1 to 63.7% for **Al1**, from 19.1 to 49.1% for **Al2**, but decreases from 20.7 to 13.0% for **Al3**. Next, the emission lifetimes (τ_em_) of **Al1–3** in CHCl_3_ were determined, which possess only slight differences and are all in the same nanosecond time regime (Figure S19 and Table S9). These lifetimes suggest that fluorescence is mainly taking place.^97^ These observations are intriguing, as they suggest a contradictory scenario in which **Al1** exhibits both the highest Φ_em_ from its singlet state and the most efficient ^1^O_2_ sensitization *via* its triplet state. Typically, ISC competes with singlet emission, making it unusual for a complex to be excellent in both processes. The photostability measurements (see below) reveal very different stabilities for the three Al(III) PSs, which is a possible explanation for the unexpected emission behavior. Compared to their lifetimes in CH_3_CN (9.6 ns for **Al1**, 5.7 ns for **Al2** and 6.4 ns for **Al3**), τ_em_ remains in the same order of magnitude.^1^

### Photostability and ^1^O_2_ Resistance

**Figure 5:**
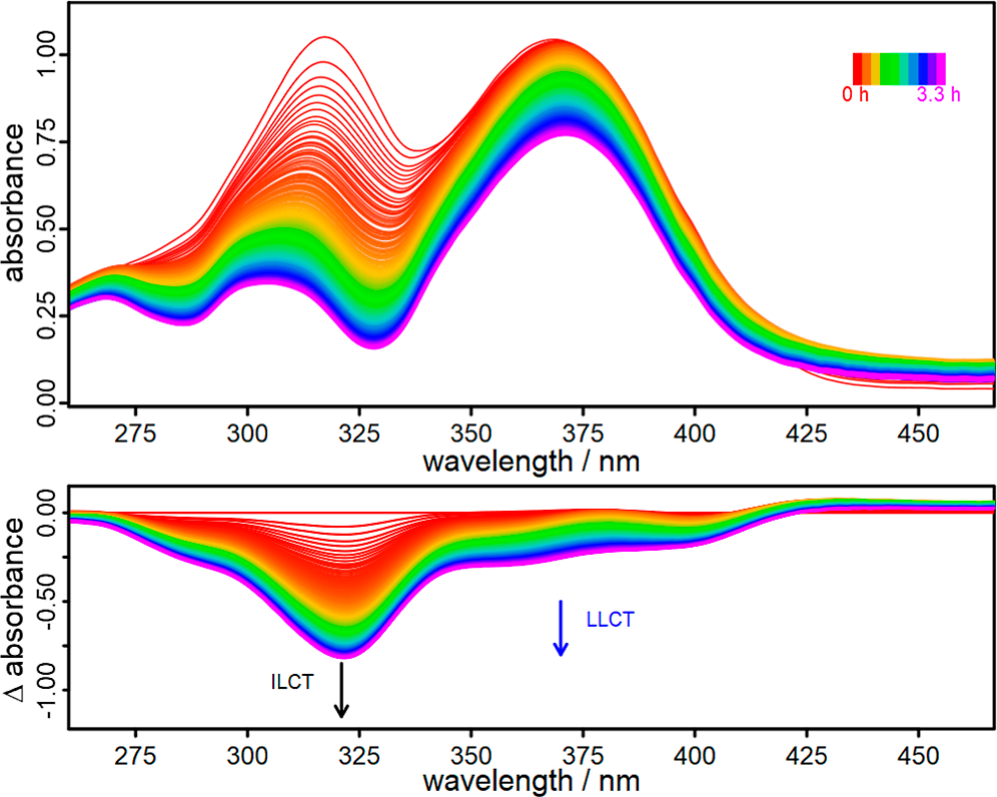
Photostability measurements of **Al1** in aerated CHCl_3_ using a 150 W Xe-lamp and a 400 nm long-pass filter.

Photostability measurements were carried out to investigate the long-term stability of the Al(III) complexes under visible light irradiation. For this issue, the absorption intensity was continuously monitored in aerated CHCl_3_ using *in situ* UV/vis spectroscopy (Figure 5). Furthermore, these experiments give information about the resistance of the Al(III) PSs toward ligand oxidation *via* reactive ^1^O_2_ and shed light on their applicability under aerated conditions.

Interestingly, the photostability measurements of **Al1** (Figure 5) reveal different results for the respective ILCT and LLCT transition bands. While the ILCT at around 321 nm decreases rapidly, the LLCT band at around 370 nm is quite stable over the course of 3 h (Figure 5). In the case of **Al1**, the given photostability is sufficient to ensure a prolonged activity with regard to photocatalysis (vide infra). For **Al2** and **Al3** (Figure S6), the photostability measurements yield a rapid decrease of both absorption bands. Furthermore, there is a strong signal arising at λ < 275 nm, which is stronger for **Al3** than for **Al2** and can be attributed to the oxidation products of the 2-pyridylpyrrolide ligands.^98^ Hence, a higher degree of ligand-methylation results in a lower resistance to ^1^O_2_ and again **Al1** is superior to the other complexes in this case. This could be the reason why **Al1** produces more ^1^O_2_ during the rather long emission measurements (Figure 2).

These observations are consistent with the literature, as ^1^O_2_ exhibits strong oxidizing properties and also shows remarkable regio- and chemoselectivity.^99^ For instance, ^1^O_2_ reacts more easily with dialkyl-substituted furans than with monoalkyl-substituted furans.^100^ These literature results also support our findings for **Al1** described above.

Moreover, photostability measurements under deaerated conditions were performed and also reveal different conversion rates for the two absorption bands (see Figure S7). For **Al1**, the LLCT band remains largely unchanged, while the ILCT band decreases with time, suggesting the formation of degradation products capable of absorbing in the LLCT range. However, for **Al2** and **Al3**, the photodegradation in the absence of oxygen is much faster than in aerated conditions and both bands exhibit a rapid decrease. This may explain the lower emission intensities and quantum yields for **Al2** and **Al3** compared to **Al1**. In the case of **Al3**, degradation products seem to be formed after prolonged excitation, which absorb at around 375 nm. This results in a new signal at longer irradiation times (see Figure S7), which is not observed under aerated conditions. However, the different photostabilities and degradation mechanisms under deaerated and aerated conditions are not fully understood and require further investigation.

### Photocatalytic Oxidation of DPF Using ^1^O_2_

**Figure 6:**
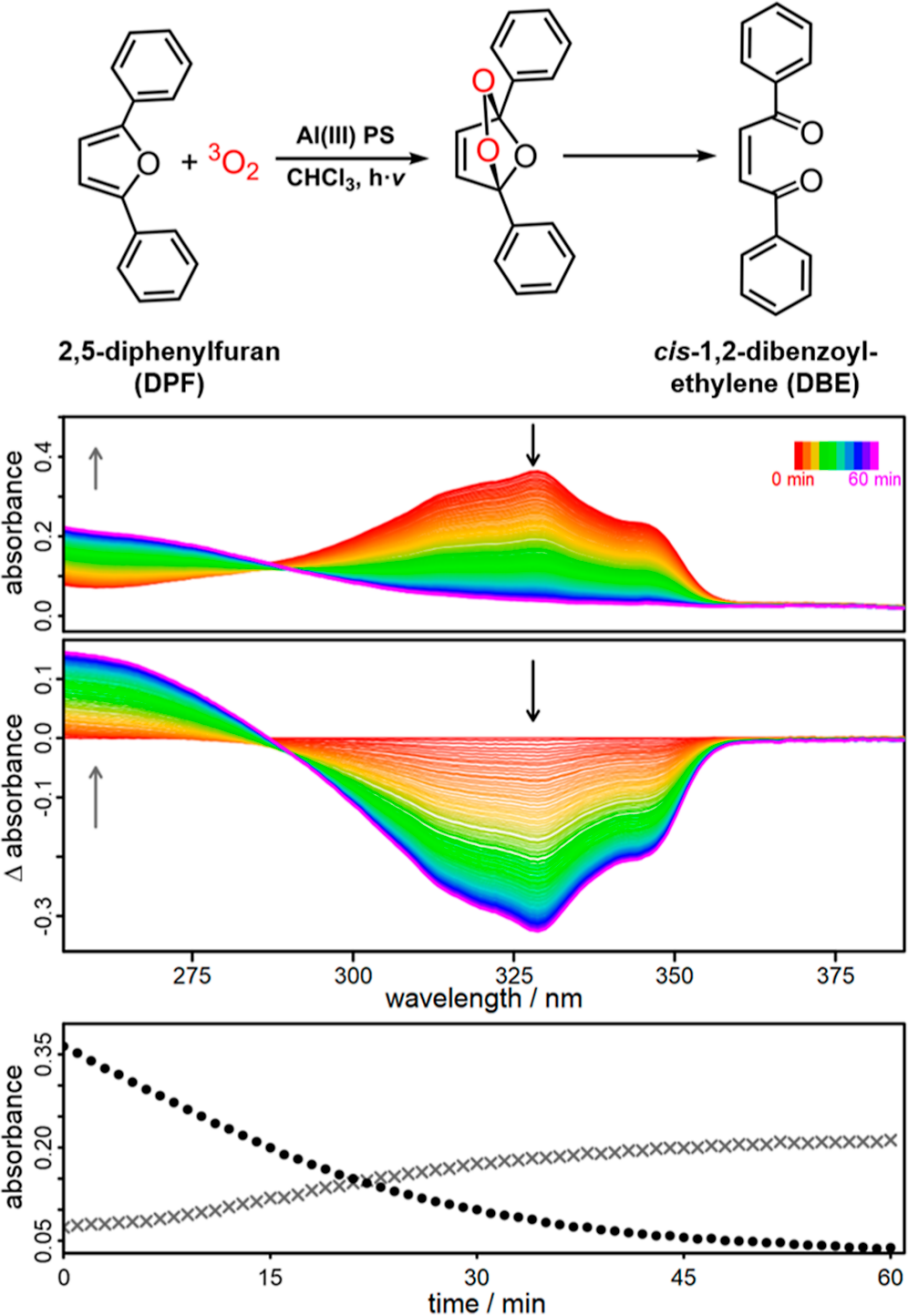
Reaction mechanism for the photocatalytic oxidation of DPF to DBE by ^1^O_2_ (top) via the intermediate endoperoxide.^72^*In situ* UV/vis absorption spectra (top spectra), the differential plots (middle spectra) and the kinetic plots (bottom) of DPF depletion at 328 nm (black dots) and DBE formation at 260 nm (gray crosses). The reaction was driven in aerated CHCl3 using **Al1** as PS (*c* = 0.95·10^–6^ M) and the molar ratio of PS/DPF was set to 1:10. A 150 W Xe-lamp equipped with a 400 nm long-pass filter was used.

Due to the previously mentioned regio- and chemoselectivity of ^1^O_2_, it is possible to select a target substrate with a much higher affinity for ^1^O_2_, such as DPF.^99,100^ It is known that DPF is easily oxidized by ^1^O_2_ to generate an intermediate endoperoxide, which then irreversibly forms DBE (Figure 6).^32,72,101^ This conversion can be conveniently observed by *in situ* UV/vis spectroscopy. During photocatalysis, the DPF absorbance at 328 nm decreases (DPF consumption), while the DBE absorbance at 260 nm increases (DBE formation).^16,19^ Consequently, the DPF oxidation not only provides a model reaction for the usage of ^1^O_2_, but also provides an indirect approach of detecting the ^1^O_2_ produced by the Al(III) PS.

After 1 h of irradiation with visible light (λ > 400 nm), a significant amount of DPF was converted to DBE for all three Al(III) complexes (Figures 6 and S20–S22). To estimate the initial singlet oxygen efficiency, the rate constant *k* is determined for all three Al(III) PSs and several reference compounds for comparison (Table 2).

**Table 2:** Initial Rate Constants *k* (First 50 Min) for the Photocatalytic Conversion of DPF Using **Al1–3** and Several Reference Complexes for Comparison

PS	*k*/min^–1^
**Al1**	0.043
**Al2**	0.030
**Al3**	0.016
**Alq3**	0.020
**CuPS**	0.029^a^
**PN**	0.042^a^
**RuPS**	0.004

a First 15 min only, because after that the conversion rate drops drastically due to decomposition.

**Figure 7:**
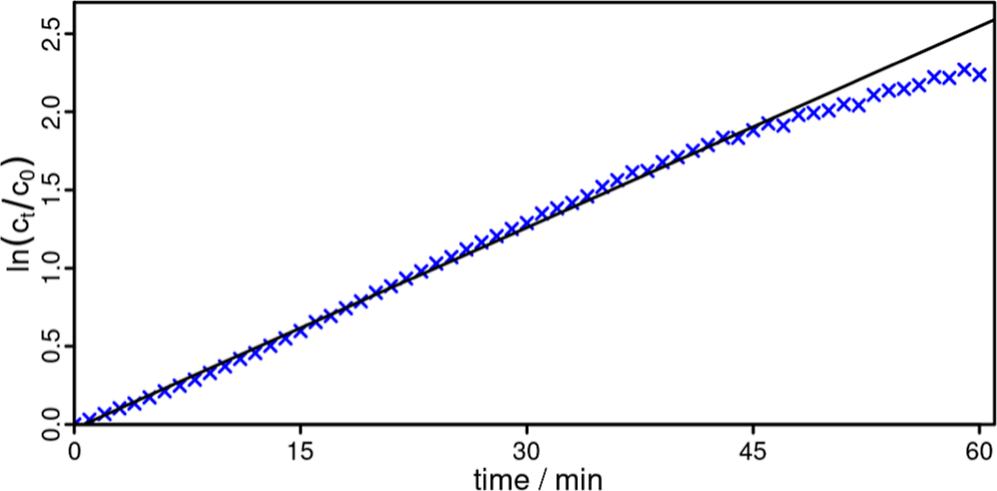
DPF conversion of **Al1** in the first hour (blue crosses) and first order fit (black) with ln(*c*_*t*_/*c*_0_) per time. The linear fit is given and the slope is estimated to be *k*_**Al1**_.

The first order kinetic fits provide rates of 0.043 min^–1^ for **Al1** (Figure 7), 0.030 min^–1^ for **Al2** and 0.016 min^–1^ for **Al3** (Figures S20–S22). Consequently, **Al1** has the highest initial rate as well as the best photostability. This is also consistent with the ^1^O_2_ emission measurements above (highest intensity). However, also **Al2** and even **Al3** enable a rather efficient DPF conversion (see Figures S21 and S22), although **Al3** showed no obvious ^1^O_2_ emission in CDCl_3_ (Figure 2). This seeming contradiction can be explained by the fact that **Al3** is the least stable complex and that the required measurement time of the ^1^O_2_ emission is quite long (0.5 s per data point). Due to the long, continuous irradiation, **Al3** is probably oxidized or decomposed before the ^1^O_2_ emission is detected. In the presence of DPF, a good ^1^O_2_ quencher,^68,101^ it is more easily oxidized than **Al3**, thus allowing the indirect detection of ^1^O_2_. As a result, DPF conversion is observed at low rates (Table 2).

Finally, to compare the photocatalytic efficiency of the Al(III) complexes, the reference compounds **Alq**_**3**_, [Cu(bcp)(xant)]PF_6_ (**CuPS**, bcp = bathocuproine and xant = xantphos) and perinaphthenone (**PN**, Figure S13) were tested under identical conditions. The Cu(I) complex exhibits a comparable conversion rate within the first 15 min, which then decreases rapidly (Figure S24), while **Alq**_**3**_ maintains its activity over time (Figure S23). However, **Alq**_**3**_ exhibits a slower conversion rate when compared to **Al1** or **Al2** (Table 2). Surprisingly, for the reference compound **PN**, which is known to have a singlet oxygen yield close to unity,^102^ a lower photoactivity can be observed over time (Figure S28). In addition, [Ru(bpy)_3_]Cl_2_·6H_2_O (**RuPS**, bpy = 2,2′-bipyridine) was used as a further reference for catalysis purposes, because this complex is still heavily used in photocatalysis.^8,24^ While this noble metal-based reference compound seems to have a good stability under these conditions, it is much slower in the conversion of DPF (0.004 min^–1^, Figures S25 and S27).

### Recycling Experiments and Long-Term Catalysis

To make photocatalysis more interesting for large-scale or industrial applications, a first step is to validate the long-term applicability and reproducibility of the system. To this end, recycling experiments were conducted to demonstrate the full potential of these Al(III) complexes. For this purpose, a fresh excess of DPF was added to the already irradiated cuvette without renewing the Al(III) PS. This procedure was repeated twice for all three complexes, each time after 1 h of irradiation (Figures S20–S22).

The recycling experiments yielded successful results for all three Al(III) complexes (see Section S6). The conversion increased significantly upon addition of fresh DPF until a point of low DPF concentration, confirming our hypothesis regarding the higher chemoselectivity of ^1^O_2_ toward DPF. Furthermore, the reference complex **CuPS** is again less active (Figure S24), which is consistent with the lower conversion rate (Table 2). Here, the addition of fresh DPF does not enhance the conversion, which means that **CuPS** is generally less active and cannot compete with the performance of the Al(III) PSs. The **Alq**_**3**_ complex showed decreased conversion rates after just two DPF additions, although better than **CuPS**, but worse than **Al1–3** (Figure S23). In the control experiments without a PS or light, only minimal DPF conversion was observed (Figure S26).

**Figure 8:**
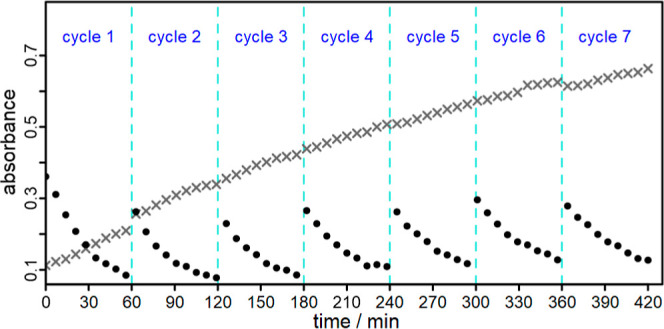
Recycling experiment for the photooxidation of DPF using **Al1** in CHCl_3_. The kinetic plots were derived from *in situ* UV/vis measurements and show the depletion of DPF at 328 nm (black dots) and the DBE generation at 260 nm (gray crosses). An 8.3-fold excess of DPF (with respect to the PS) was added after each hour of irradiation.

In a next step, a long-term experiment (7 cycles, 7 h) was carried out to test the limits of **Al1**, which is the most photostable and promising PS (Figure 8). DPF was freshly added after 1 h each. As a result, a continuous conversion of DPF to DBE was found over a period of 7 h (Figure 8). Only after 5 cycles (5 h) there is a slight decrease in DPF conversion, which is most likely due to the slow oxidation of **Al1**. Although DPF is a much better scavenger for ^1^O_2_ than **Al1**, the high conversion leads to a situation where only little amount of DPF is left, increasing the probability for ligand oxidation.

Nevertheless, **Al1** showed the best results of all studied PSs. It has the best photostability, the highest initial rate of DPF conversion within the first hour and also mostly maintains its conversion rate over several DPF additions. This is also in accordance with our findings mentioned above, where the lower degree of ligand methylation causes better energy transfer properties and a higher photostability.

### Photosensitized Isomerization of (*E*)-Stilbene

Finally, the photocatalytic isomerization of (*E*)- to (*Z*)-stilbene was performed to investigate **Al1–3** in the frame of a second energy transfer reaction to demonstrate their versatility.^12,13,30^ Alkene derivatives such as stilbenes are synthesized both naturally and synthetically and represent a class of compounds with a wide range of bioactive properties (e.g. retinal in the human photoreceptors).^13,103,104^ There, the stereochemical configuration of the molecule is of utmost importance.^13,104^ The (*E*)- to (*Z*)-isomerization of alkenes using light is a mild and powerful method to manipulate C(sp^2^)=C(sp^2^) bonds.^13,105^ In this case, (*E*)-stilbene is excited to its triplet state by energy transfer from an excited PS molecule, weakening the torsional barrier within the double bond.^13,105^ Subsequent relaxation of this more excited state with a bond order of one within the respective ππ* state then leads to the formation of either (*E*)- or (*Z*)-stilbene. While the ground state (S_0_) of (*Z*)-stilbene is slightly higher in energy than the S_0_ of (*E*)-stilbene, the sensitization of (*Z*)-stilbene is less effective.^13,30^ This results from the lower triplet energy level (T_1_) of the (*E*)-isomer (206 kJ·mol^–1^) compared to the T_1_ energy of (*Z*)-stilbene (227 kJ·mol^–1^). Therefore, the (*Z*)-isomer is produced until a photostationary state is reached, where the concentration of each species form an equilibrium.^13,104,105^ Thus, yields below 100% and slow overall conversion rates are expected.

**Figure 9:**
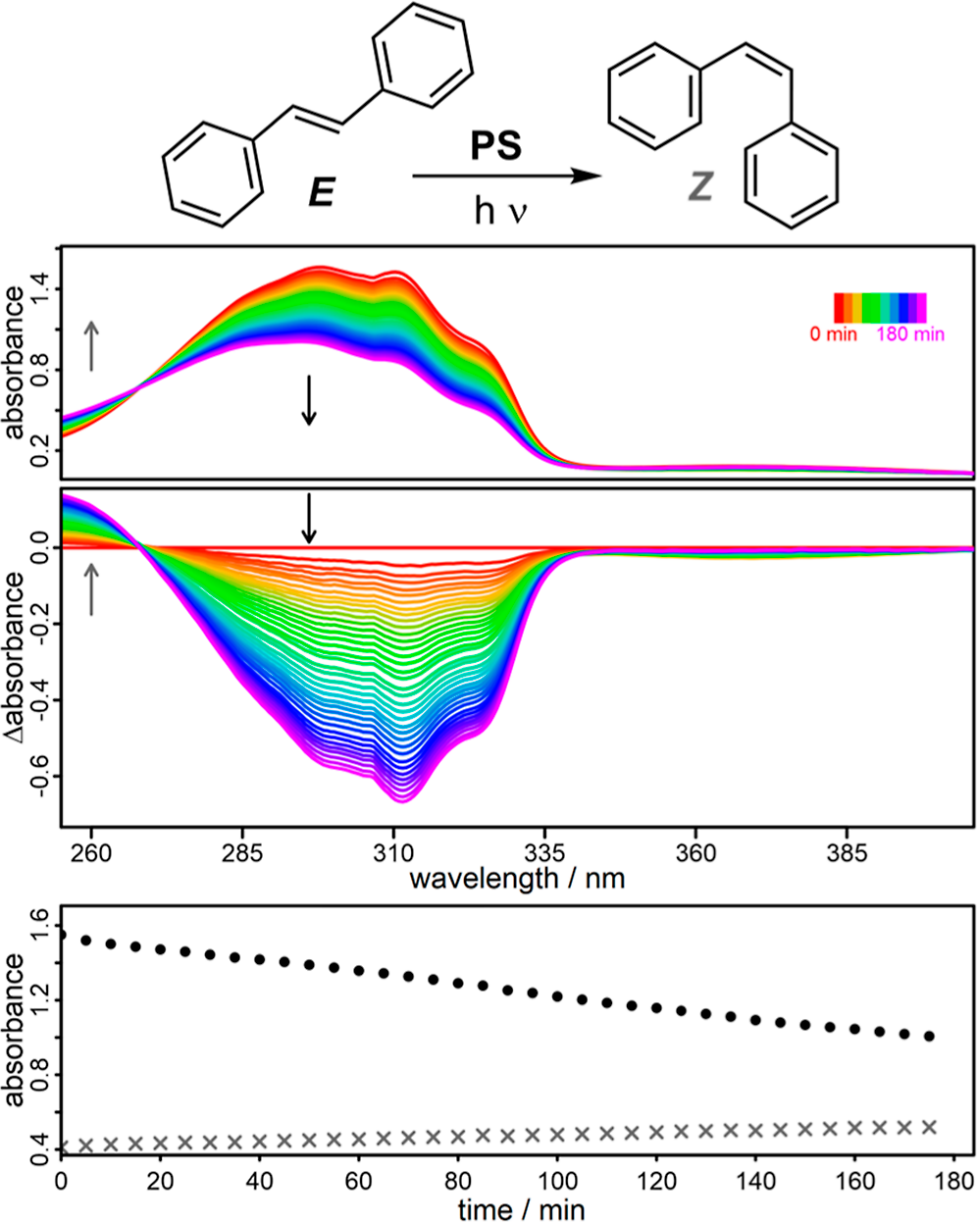
Reaction scheme for the photocatalytic isomerization of (*E*)- to (*Z*)-stilbene (top) using **Al1** in CHCl_3_ under inert conditions. *In situ* UV/vis absorption spectra (top spectra), differential plots (middle spectra) and kinetic plots (bottom) of *E*-stilbene depletion at 296 nm (black dots) and (*Z*)-stilbene generation at 260 nm (gray crosses). A 405 nm power LED (∼10.7 W) and a 400 nm long-pass filter was used. The PS concentration was 7.8 × 10^–6^ M and the molar ratio of PS to (*E*)-stilbene was adjusted to 1:25.

Once more, *in situ* UV/vis spectroscopy is used as a powerful tool to monitor the photocatalytic isomerization of (*E*)- to (*Z*)-stilbene by the characteristic absorption of the two different isomers [(*E*)-stilbene λ_max_ = 296 nm and (*Z*)-stilbene λ_max_ = 260 nm].^106^ Subsequently, **Al1–3**, **Alq3** and **CuPS** were successfully investigated (Figures 9 and S28), but with different activities. The same trends emerged as for the photocatalytic generation of ^1^O_2_ with **Al1** showing the best conversion of (*E*)- to (*Z*)-stilbene. The higher the degree of ligand methylation, the worse the conversion (**Al1** > **Al2** > **Al3**), which can be explained by the faster decomposition of **Al2** and **Al3** under deaerated conditions. Although **CuPS** initially shows a similar efficiency as **Al1**, it deactivates faster than **Al1**, reaching a plateau after the first 90 min, whereas **Al1** is active for 3 h. **Alq**_**3**_ also exhibits a lower activity in this isomerization process, making **Al1** to the best PS in this study in all areas. The control experiments revealed that light and a PS are required for the photocatalytic isomerization of (*E*)- to (*Z*)-stilbenes (Figure S29).

## Conclusions

For the first time, a series of three Al(III)-based PSs was systematically investigated in light-driven energy transfer reactions, i.e. the photocatalytic generation of singlet oxygen and the (*E*) to (*Z*)-isomerization of alkenes. The three homoleptic Al(III) complexes are derived from the 2-pyridylpyrrolide ligands, which differ in the number of their methyl substituents per ligand (**Al1** = no substituents, **Al2** = one methyl group and **Al3** = two methyl groups).

Along with the photocatalytic studies, which were carried out in chloroform, the main photophysical properties were determined in the same solvent. The spectroscopic measurements revealed that **Al1** has the highest emission quantum yield (64%) and the longest emission lifetime (8.7 ns) compared to **Al3** with the worst results (13% and 7.2 ns). This already indicated a strong impact of the methyl substituents and that a lower degree of methylation is advantageous with respect to the photophysical properties of the photosensitizer.

This trend also holds true for the formation of singlet oxygen upon light irradiation of the Al(III) complexes, which was directly detected by its characteristic NIR emission at around 1276 nm. This means that **Al1** showed the highest emission intensity and **Al2** a medium one, while **Al3** gave no significant signal. The successful generation of singlet oxygen also confirmed the existence of a dark, but long-lived triplet excited state for **Al1–3** (2.13–1.44 μs) observed in a previous study.^1^ The result that **Al1** is both the best fluorophore (highest emission quantum yield *via* a singlet state) and the best triplet sensitizer (highest ^1^O_2_ production *via* a triplet state) is unexpected, but subject to further investigation.

The generated ^1^O_2_ was then applied in the photocatalytic oxidation of DPF to *cis*-1,2-dibenzoyl-ethylene. The photooxidation of DPF also served as an indirect detection of singlet oxygen and was continuously monitored by *in situ* UV/vis spectroscopy. As a result, **Al1** provided the highest initial conversion rate (*k* = 0.043 min^–1^ within the first 50 min), which was similar to that of the internal standard **PN**. The activity of **Al1** also clearly exceeds that of several reference complexes such as [Ru(bpy)_3_](PF_6_)_2_. **Al2** had the second highest (0.030 min^–1^) and **Al3** the lowest conversion rate (0.016 min^–1^), but still in the same order of magnitude as the **Alq**_**3**_ reference complex (0.020 min^–1^). This order of activity was rather surprising, as **Al3** showed the best performance in an earlier study in the photocatalytic reduction of CO_2_ to CO using a reductive quenching mechanism.^1^ There, the improved absorptivity and reducing power caused by the additional methyl groups were identified as the most important advantages, but play a less important role in energy transfer reactions.

A decrease in the photocatalytic ^1^O_2_ production over time was observed for all PSs, but was lowest for **Al3** and **Alq**_**3**_. Nevertheless, especially **Al1** is quite stable for at least 1 h, illustrating the high potential of these earth-abundant complexes under these harsh reaction conditions. In combination with its high activity, **Al1** was successful in a photocatalytic long-term test (7 h), in which this complex was recycled 7 times with only a slight decrease in the conversion rate.

Beyond ^1^O_2_ generation, the Al(III) complexes were examined in the photosensitized isomerization of (*E*)- to (*Z*)-stilbene, illustrating their broad applicability. Again, **Al1** demonstrated the highest efficiency among all investigated PSs. **Al1** maintained its activity even after 3 h, while **Alq**_**3**_ was ineffective in facilitating the isomerization of (*E*)-stilbene.

In conclusion, this study proved the versatility of Al(III)-based PSs and successfully uncovered essential structure–activity relationships. **Al1–3** can be used not only in photoredox catalysis involving electron transfer, but also in energy transfer reactions. In the latter context, **Al1**—without the additional methyl substituents—is clearly superior as it combines the best stability with the highest activity. Based on these positive catalytic results, further studies are currently underway to investigate the light-driven reaction mechanism, i.e. the triplet reactivity as well as the energy transfer, in more detail. Future studies will include other photocatalytic applications such as the reductive dehalogenation of arenes to bring the field of solar energy conversion and earth-abundant PSs one step further.

## Experimental Details

### Steady-State Absorption Spectroscopy

Steady-state UV/vis absorption spectra were recorded with a JASCO V-770 spectrophotometer. All samples were measured in solution in a standard 1 cm quartz glass cuvette. The bandwidths were set to 1 nm in the UV/vis region, the continuous scan rate was set to 400 nm/min and the data interval was chosen to be 1 nm.

### Time-Dependent In Situ Absorption Spectroscopy

*In situ* measurements of time-dependent UV/vis absorption spectra during (photo)chemical reactions (e.g., photostability, isomerization, DPF catalysis) were recorded with an Avantes StarLine AvaSpec-ULS2048CL-EVO-RS spectrophotometer connected to an Avantes AvaLight-DH-S-BAL light source via fiber-optic cables. If not stated otherwise, the integration time was set to 1.25 ms and each spectrum was averaged over 100 measurements.

### Visible Emission Measurements and Quantum Yields

To determine the vis emission properties, a Horiba FluoroMax Plus was used. As for the detector, a R928P photon counting PMT was used for the visible emission. The emission was measured under Argon atmosphere and all optical densities of the complexes were adjusted to 0.1 at the excitation wavelength. Thus, no oxygen quenching or self/-reabsorption takes place.

For the quantum yield calculation, eq 1 was used^1^
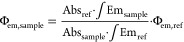
1

The chosen reference Alq_3_ has a known emission quantum yield of 11.6% in deaerated acetonitrile.^96^ The error of the quantum yields is estimated to be 10%.

### NIR Emission Measurements

To determine the NIR emission properties, a Horiba FluoroMax Plus was used. As for the detector, a DSS-IGA020L InGaAs solid state detector for the NIR emission. The detector was cooled with liquid nitrogen for at least 30 min before use. All optical densities of the complexes were adjusted to 0.1 at the excitation wavelength and thus, no self/-reabsorption takes place. Air-saturated solvents were used for NIR emission measurements (^1^O_2_ sensing).

### Emission Lifetime and Streak Camera Measurements

Streak images and corresponding emission lifetimes were obtained using a HAMAMATSU Universal Streak Camera C10910 coupled with a Kymera 328i-A spectrograph by ANDOR at 21 °C and a HAMAMATSU digital camera (C13440). A 373 nm pulsed laser (model: M10306-27-PLP-10 Laser diode head 380 nm) with a pulse duration of 42 ps and a peak power of 21 μW and a repetitions rate of 4 MHz was used as the excitation source. The samples were solved in chloroform under inert conditions with an optical density of about 0.1 at the excitation wavelength.

### Time-Dependent Absorption Spectroscopy

Time-dependent UV/vis absorption spectroscopy was used to record *in situ* absorption spectra during photocatalytic measurements. This was done using an Avantes StarLine AvaSpec-ULS2048CL-EVO-RS spectrophotometer and an Avantes AvaLight-DH-S-BAL light source. The light source was connected to the spectrophotometer via fiber-optic cables. The data acquisition time was set to 1.25 μs and an average of 100 measurements was taken for the final spectra.

### Photooxidation of DPF

Three solutions were prepared first, yielding a 1.9·10^–4^ M solution of DPF (solution A), a 1.9·10^–5^ M solution of DPF (solution B) and a 1.9·10^–6^ M solution of PS (solution C) respectively. Now, 1.3 mL of B and C were added into a cuvette, as well as a micro stirring bar. During catalysis, stirring was set to 800 rpm. In the case of recycle experiments, 130 μL of solution A were added to the cuvette mixture after 1 h. In the extended recycle experiment for **Al1**, the added volume was adjusted to 100 μL to prevent cuvette overflow. This modification was crucial to ensure that a sufficient number of recycling steps could be performed. A 150 W Xe-lamp by Quantum Design was used as light source and a 400 nm long-pass filter was applied to prevent any UV-light to illuminate the solution.

### Isomerization Reactions

The stock solutions of PS (7.8 × 10^–5^ mol/mL^3^) and *E*-stilbene (6.5 × 10^–4^ mol/mL^3^) in chloroform under inert conditions were prepared in separated Schlenck tubes under constant argon flow using Schlenk technique.^107^ The spectroscopic cuvette (*V* = 4 mL; *d* = 1.0 cm) with a stirring bar inside was placed in a Schlenk tube and evacuated and flooded with argon three times. Afterward 2.7 mL of degassed chloroform, 0.3 mL of PS stock solution and 0.1 mL of *E*-stilbene stock solution were added into the cuvette. The final mole ratio PS/*E*-stilbene was 1:25 for each measured sample. The cuvette was placed inside the Avantes spectrometer (Avantes AvaSpec-ULS2048CL-EVO) above a magnetic stirrer (600 rpm) and a UV/vis spectrum was recorded every 5 min averaging 100 measurements every 1.75 ms. A power 405 nm LED was used as a light source (spectrum of the LED is given in Figure S1) and additionally a 400 nm long-pass filter was placed in the light path. The LED was set to *U* = 14.25 V & *I* = 0.75 A, resulting in a power input of approximately 10.7 W.

### Computational Details

If not stated otherwise, all quantum chemical calculations determining structural and electronic properties were performed using the Gaussian 16 program.^108^ Equilibrium geometry of **Al1**, **Al2 Al3** within the singlet ground state (S_0_) as well as within the triplet ground state (T_1_) were obtained at the density functional level of theory (DFT) by means of the B3LYP XC functional.^86,87^ Therefore, the previously optimized structures of **Al1**–**Al3** as obtained in acetonitrile were utilized as initial guess geometries, respectively.^1^ The def2-SVP basis set was applied for all atoms.^88,89^ Effects of interaction with a solvent (chloroform: ε = 4.7113, *n* = 1.444) were considered by the solute electron density (SMD) variant of the integral equation formalism of the polarizable continuum model.^109,110^ D3 dispersion correction with Becke–Johnson damping was included in all DFT calculations.^111^ A subsequent vibrational analysis was carried out for each optimized ground state structure to verify that a minimum on the potential energy (hyper-)surface (PES) was obtained.

Excited state properties such as excitation energies, oscillator strengths and electronic characters were calculated within all singlet ground state equilibrium structures at the TDDFT level of theory. Therefore, the 50 lowest singlet excited states were calculated, while the same computational protocol (B3LYP, basis set and solvent model) was applied as for the preceding DFT calculations. The nonequilibrium procedure of solvation was used for the calculation of the excitation energies within the Franck–Condon region, which is well adapted for processes where only the fast reorganization of the electronic distribution of the solvent is important. Previously, this computational protocol was shown to provide an unambiguous description (with respect to the long-range corrected CAM-B3LYP^112^ and the B3LYP10 functional—a functional based on B3LYP with 10% exact exchange) of the ground and excited state properties for this class of Al(III) complexes.^1,67^

Additionally, scalar-relativistic TDDFT simulations were carried out utilizing Orca 5.0.3^113^ with the Douglas–Kroll–Hess (DKH) approach to assess prominent pathways for ISC of **Al1**, **Al2** and **Al3** within the previously optimized S_0_ structures. Notably, the herein applied DKH method is computationally slightly less demanding than the zeroth-order regular approximation^114^ while both methods typically provide comparable accuracies. To this aim, the B3LYP functional and DKH-def2-SVP basis sets (with the corresponding auxiliary basis sets using autoaux) were used.^115,116^ The 50 lowest singlet–singlet and singlet–triplet excitations were obtained, while SOCs between these states and the singlet ground state were obtained at the scalar-relativistic TDDFT level of theory using the RI-SOMF(1X) method.^113,117^ Implicit solvent interactions (chloroform) were taken into consideration by means of the SMD model.^109^

## Supporting Information Available

Experimental details, additional UV/vis and emission spectra, emission lifetime measurements, details and data addressing the catalytic experiments as well as additional quantum chemical results (PDF)
