# Supplementary material for: Exploring the Potential of Al(III) Photosensitizers for Energy Transfer Reactions
Source: Inorg Chem. 2024 Aug 12;63(34):15829–40. doi: 10.1021/acs.inorgchem.4c01922 (PMC11351184; doi:10.1021/acs.inorgchem.4c01922)
Supplement: Supplementary file 1 — ic4c01922_si_001.pdf [file ic4c01922_si_001.pdf]

Supporting Information

Exploring the Potential of Al(III) Photosensitizers for Energy Transfer Reactions

Volkan Caliskanyürek[1], Anastasiia Riabchunova[1], Stephan Kupfer[2], Fan Ma[3], Jia-Wei Wang[3,*], and Michael Karnahl[1,*]

[1] Department of Energy Conversion, Institute of Physical and Theoretical Chemistry, Technische Universität Braunschweig, Rebenring 31, 38106 Braunschweig, Germany.

[2] Institute of Physical Chemistry, Friedrich Schiller University Jena, 07743 Jena, Germany.

[3] School of Chemical Engineering and Technology, Sun Yat-sen University, Zhuhai 519082, China.

Corresponding Authors

E-Mail: wangjw89@mail.sysu.edu.cn (Jia-Wei Wang)

E-Mail: michael.karnahl@tu-bs.de (Michael Karnahl)

Table of Content

1 Further Experimental Details........2

2 Singlet Oxygen Experiments in Different Solvents........3

3 Absorption Properties of Al1-3........5

4 Quantum Chemical Results........7

5 Molecular Structures and Purity of the Reference Compounds........17

6 Emission Properties of Al1-3 and Alq_3........20

7 Photooxidation of 2,5-Diphenylfuran........22

8 DPF Conversion Rates for Al2, Al3, Alq_3, PN and CuPS........27

9 Isomerization of (*E*)-Stilbene........29

10 References........31

1 Further Experimental Details.

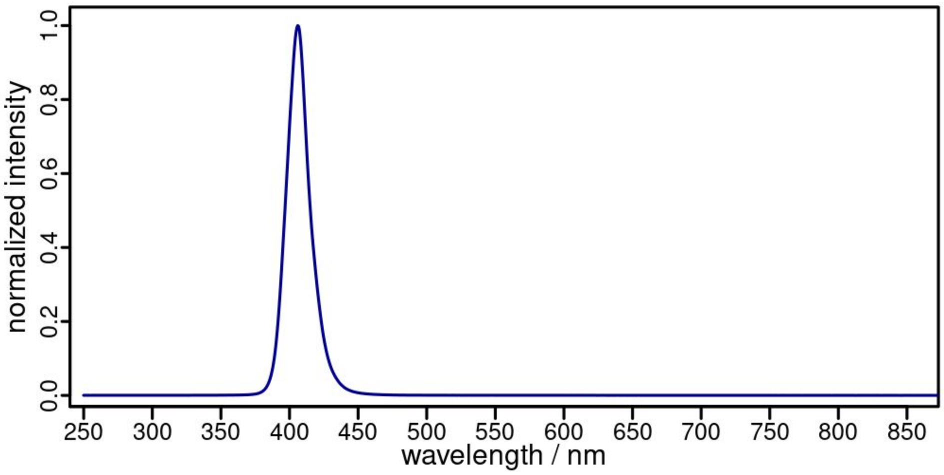

Figure S1. Emission spectrum of the power 405 nm LED light source.

Inert conditions. Inert conditions or deaerated solvents refer to experimental setups where freshly distilled and dried solvents were used in combination with Schlenk techniques and argon as an inert gas. This procedure is used to prevent any interaction with atmospheric oxygen or moisture.

2 Singlet Oxygen Experiments in Different Solvents

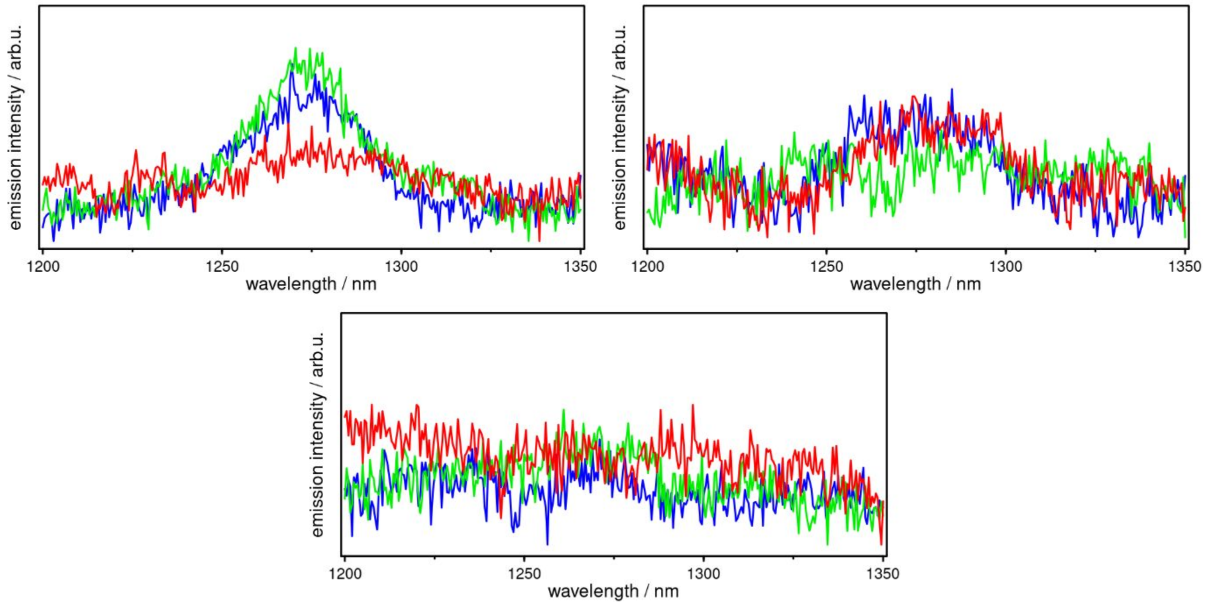

Figure S2. $^1O_2$ emission measurements in aerated chloroform (top left), dichloromethane (top right) and tetrahydrofuran (bottom) of **Al1** (blue), **Al2** (green) and **Al3** (red). OD = 0.1 for λ_{exc}. λ_{exc} are listed in Table S1.

To determine the $^1O_2$ quantum yield $\Phi_{^1O_2}$, a sufficiently intense and well resolved $^1O_2$ emission signal is required, which in the case of these Al(III) complexes is only available in $CDCl_3$ (see Figure S2 & S3). However, commonly used reference compounds such as perinaphthenon (**PN**) either have no reported values in $CDCl_3$ or lack stability in this solvent. The heteroleptic Cu(I) complex [Cu(bcp)(xant)]PF_6 (**CuPS**, with bcp = bathocuproine and xant = xantphos, see Figure S14) could serve as another possible reference compound. However, both **PN** and **CuPS** decompose immediately or over time when irradiated in $CDCl_3$, making them unsuitable for our needs.

Table S1. Excitation wavelengths λ_{exc} of $^1O_2$ emission measurements in various solvents (Figure S2).

Al(III)PS	$\lambda_{exc,chloroform}$	$\lambda_{exc,dichloromethane}$	$\lambda_{exc,tetrahydrofuran}$
**Al1**	369	366	366
**Al2**	379	380	380
**Al3**	400	398	398

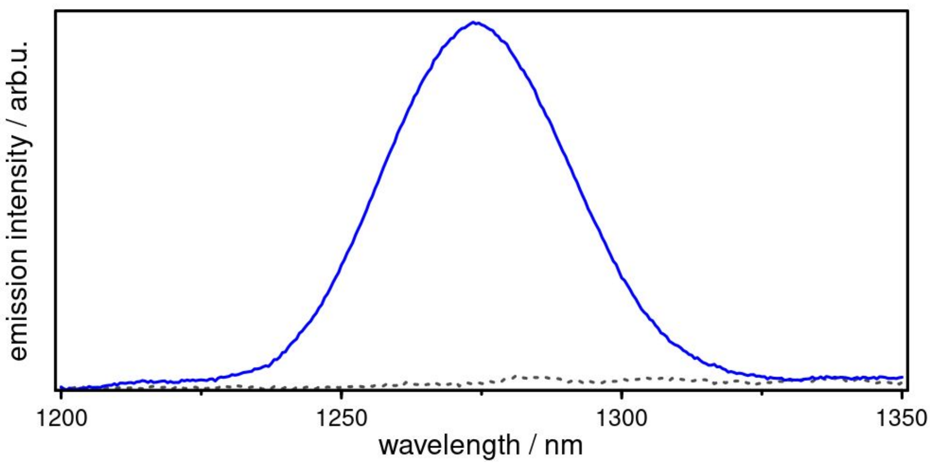

Figure S3. $^1O_2$ emission spectra with **Al1** before (blue) and after (black dotted) addition of 9,10-diphenylanthracene (DPA) in aerated, deuterated chloroform.

3 Absorption Properties of Al1-3

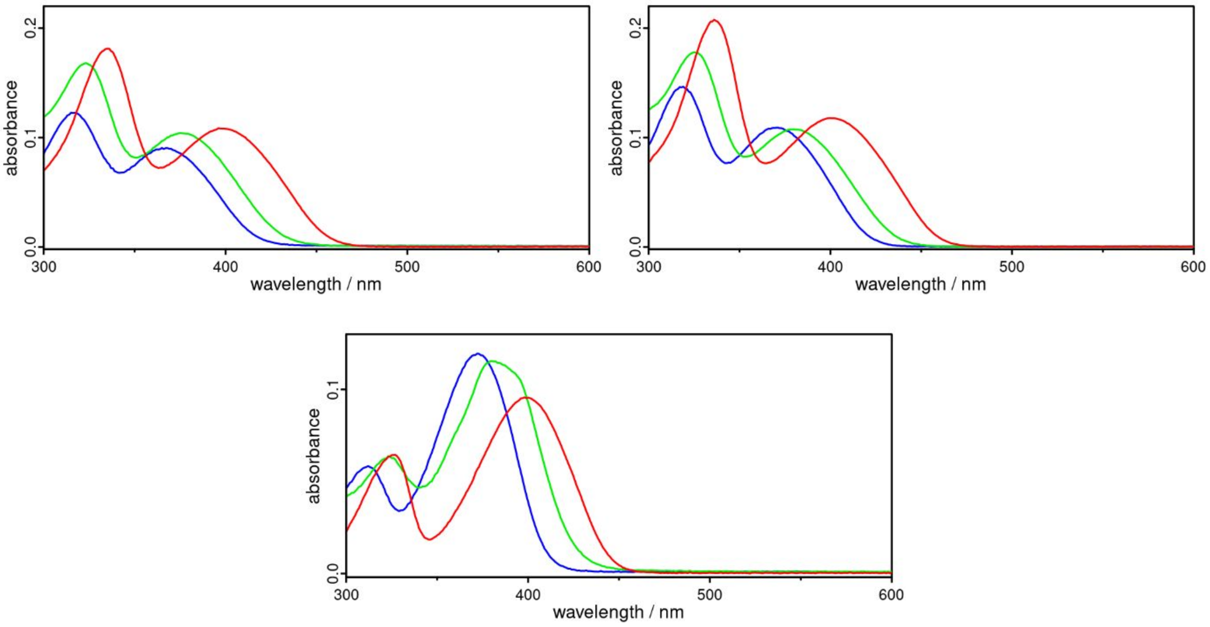

Figure S4. Absorption of **Al1** (blue), **Al2** (green) and **Al3** (red) in aerated dichloromethane (top left) and tetrahydrofuran (top right) and deuterated chloroform (bottom).

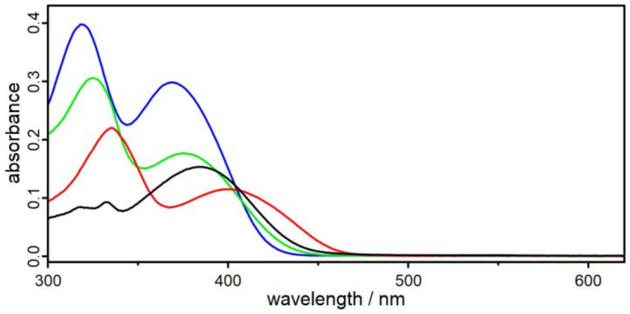

Figure S5. Absorption of **Al1** (blue), **Al2** (green), **Al3** (red) and **Alq$_3$** (black) in deaerated chloroform.

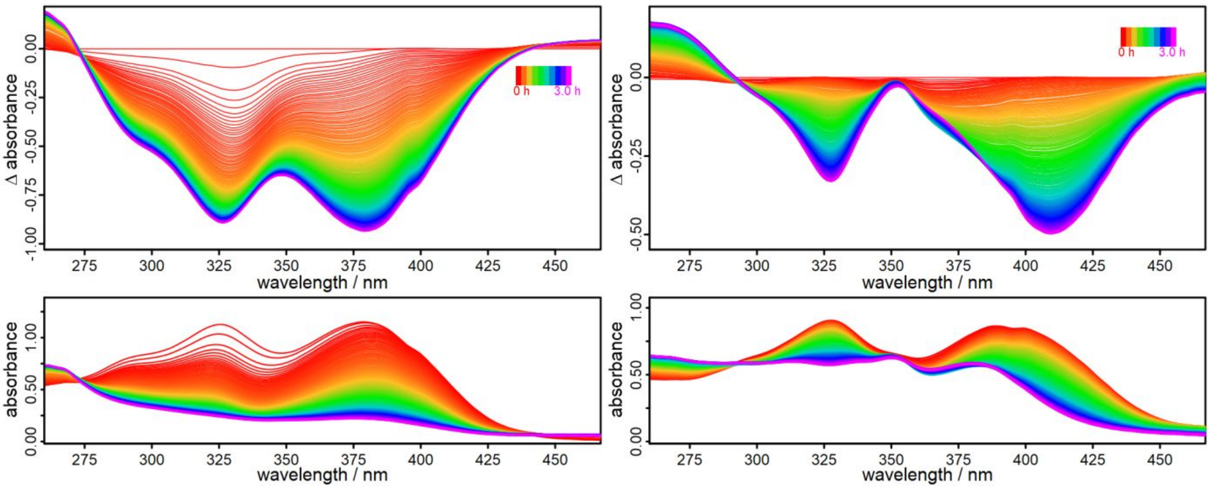

Figure S6. Photostability measurements of **Al2** (left) and **Al3** (right) in aerated chloroform. A 150 W Xe lamp with a 400 nm long-pass filter was used as excitation source.

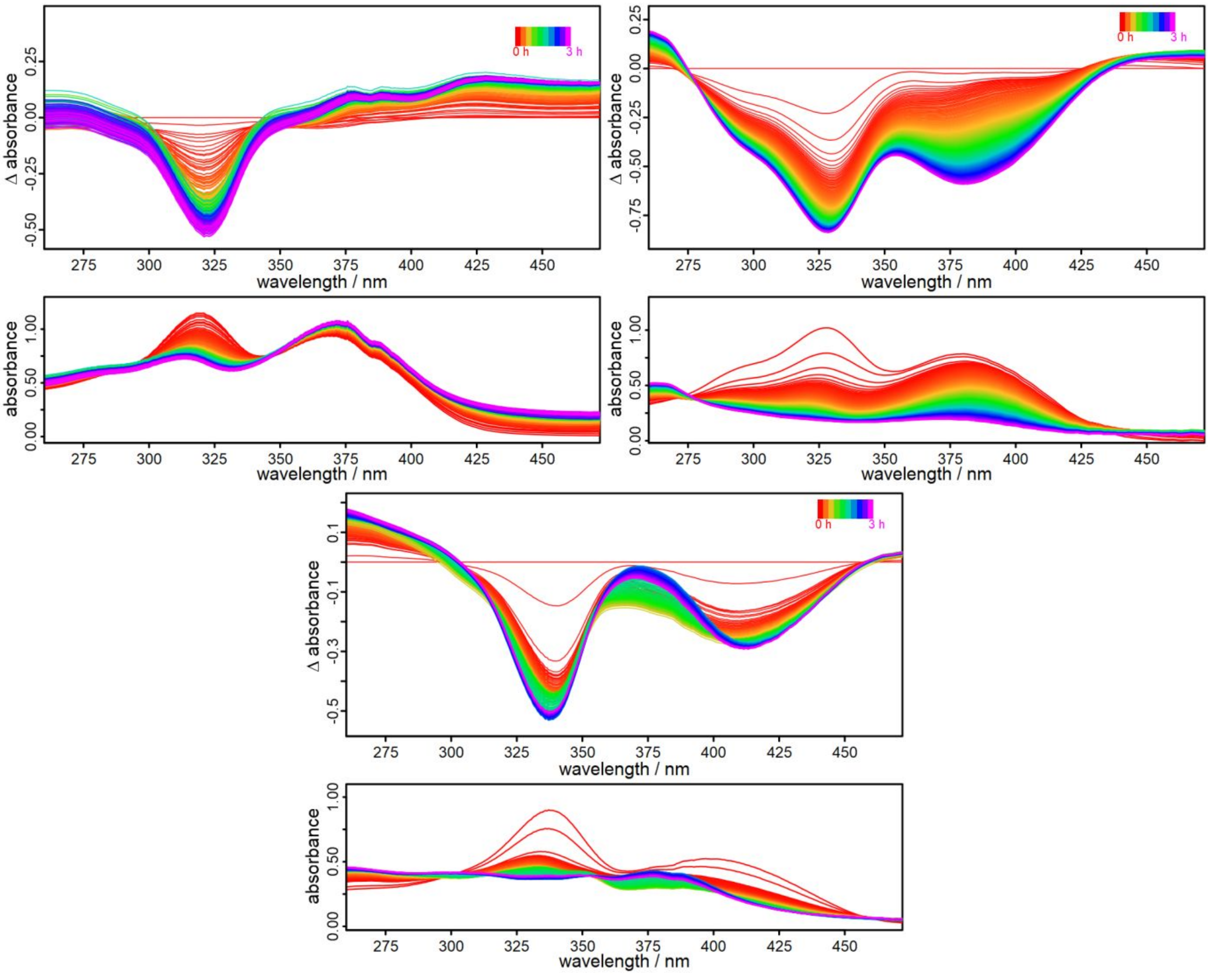

Figure S7. Photostability measurements of **Al1** (top left), **Al2** (top right) and **Al3** (bottom) in degassed chloroform. A 150 W Xe lamp with a 400 nm long-pass filter was used as excitation source.

4 Quantum Chemical Results

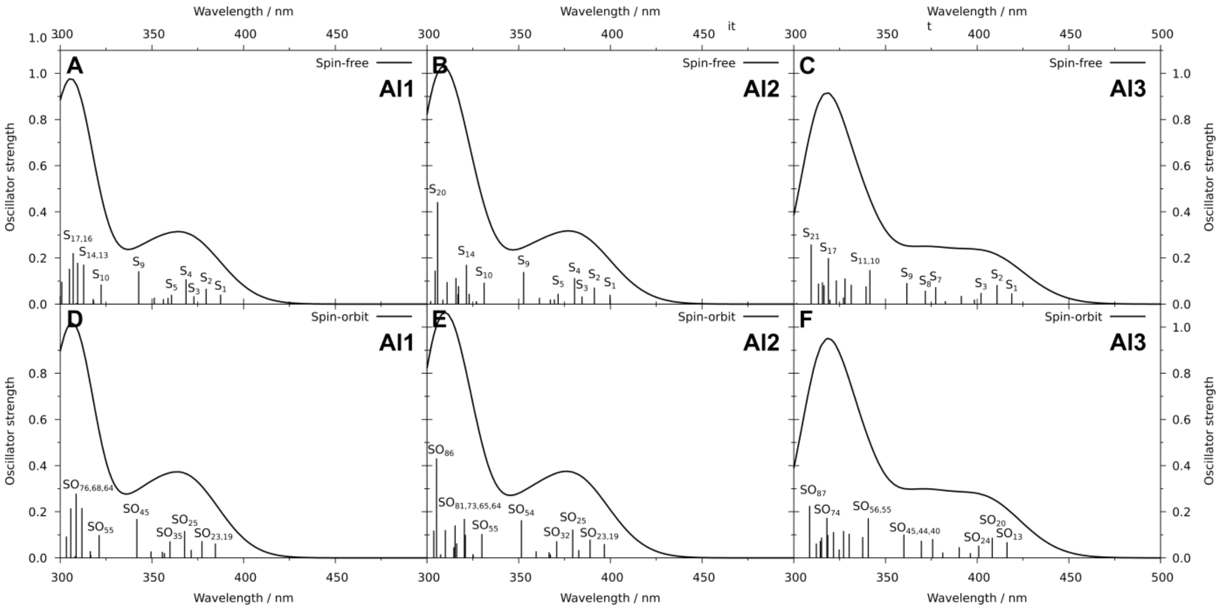

Figure S8. Spin-free and spin-orbit electronic absorption spectra of **Al1** (A,D), **Al2** (B,E) and **Al3** (C,F) as predicted at the TDDFT and scalar-relativistic TDDFT levels of theory (B3LYP/def2-SVP, chloroform). Prominent dipole-allowed electronic transitions are labelled.

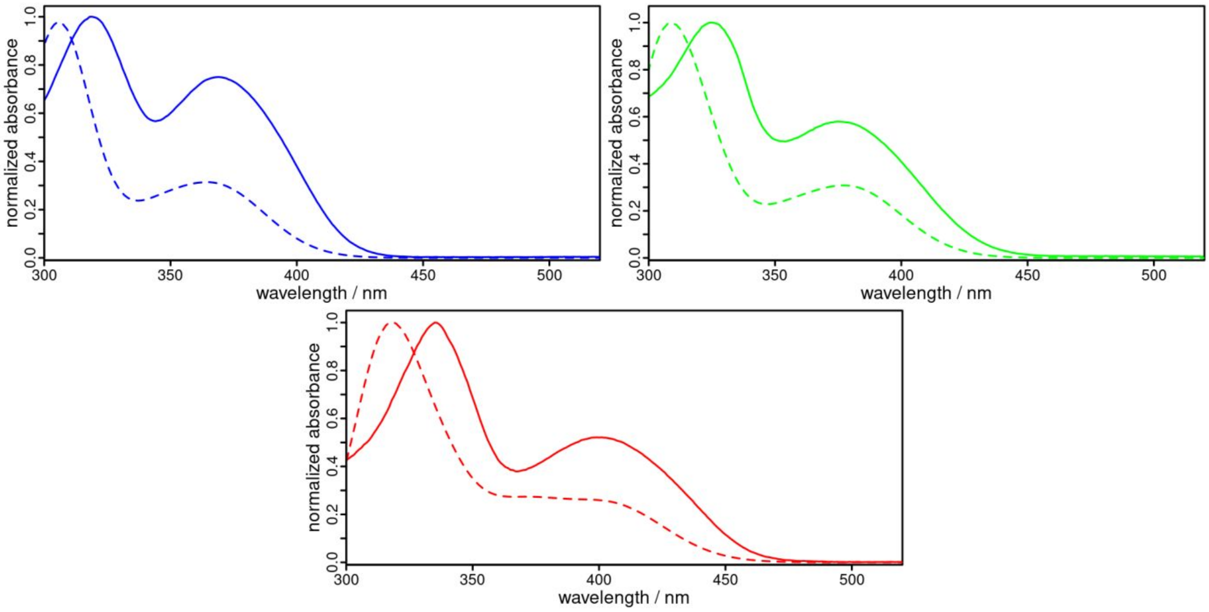

Figure S9. Experimental (solid line) and theoretical absorption (dashed line) of **Al1** (blue), **Al2** (green) and **Al3** (red) in (deaerated) chloroform.

The experimental and theoretical spectra show the same features and strong similarities in shape. The red shift with higher degree of methylation is well described by the TDDFT calculations.

Table S2. Simulated excited state properties such as excitation energies, wavelengths oscillator strengths and electronic characters of prominent dipole-allowed transitions contributing to the electronic absorption spectrum of **Al1** as obtained at (spin-free) TDDFT level of theory (B3LYP/def2-SVP) within the fully optimized singlet ground state (S_0) geometry. Implicit solvent effects were described by a polarizable continuum model (SMD, chloroform).

Transition ($S_0 \rightarrow S_n$)	Character	ΔE_n / eV	λ_n / nm	f_{0n}
S_1	LLCT	3.20	387	0.0409
S_2	LLCT	3.27	380	0.0669
S_3	ILCT	3.32	373	0.0341
S_4	ILCT	3.36	369	0.1073
S_5	ILCT	3.44	361	0.0407
S_9	ILCT	3.62	343	0.1426
S_{10}	ILCT	3.85	322	0.0853
S_{13}	ILCT	3.96	313	0.1713
S_{14}	ILCT	4.01	310	0.1793
S_{16}	LLCT	4.04	307	0.2210
S_{17}	LLCT	4.06	305	0.1532

Table S3. Simulated excited state properties such as excitation energies, wavelengths oscillator strengths and electronic characters of prominent dipole-allowed transitions contributing to the electronic absorption spectrum of **Al2** as obtained at (spin-free) TDDFT level of theory (B3LYP/def2-SVP) within the fully optimized singlet ground state (S_0) geometry. Implicit solvent effects were described by a polarizable continuum model (SMD, chloroform).

Transition ($S_0 \rightarrow S_n$)	Character	ΔE_n / eV	λ_n / nm	f_{0n}
S_1	LLCT	3.10	400	0.0405
S_2	ILCT	3.17	391	0.0720
S_3	ILCT	3.22	385	0.0332
S_4	ILCT	3.26	380	0.1126
S_5	ILCT	3.34	372	0.0446
S_9	ILCT	3.52	353	0.1391
S_{10}	ILCT	3.74	331	0.0930
S_{14}	ILCT	3.86	322	0.1703
S_{20}	ILCT	4.05	306	0.4425

Table S4. Simulated excited state properties such as excitation energies, wavelengths oscillator strengths and electronic characters of prominent dipole-allowed transitions contributing to the electronic absorption spectrum of **Al3** as obtained at (spin-free) TDDFT level of theory (B3LYP/def2-SVP) within the fully optimized singlet ground state (S_0) geometry. Implicit solvent effects were described by a polarizable continuum model (SMD, chloroform).

Transition ($S_0 \rightarrow S_n$)	Character	ΔE_n / eV	λ_n / nm	f_{0n}
S_1	LLCT	2.96	419	0.0467
S_2	ILCT	3.02	411	0.0832
S_3	ILCT	3.08	402	0.0484
S_7	ILCT	3.29	377	0.0739
S_8	ILCT	3.34	372	0.0577
S_9	ILCT	3.43	362	0.0914
S_{10}	ILCT	3.63	342	0.1476
S_{11}	ILCT	3.65	339	0.0769
S_{17}	ILCT	3.89	319	0.1993
S_{21}	ILCT	4.01	309	0.2585

The TDDFT simulations predict a switch of the electronic character from ^{3}ILCT in case of **Al1** to ^{3}LLCT for **Al2** and **Al3**. We account this observation to the gradually increased electronic density by the methyl groups at the pyrrolide rings. Therefore, it becomes energetically more favourable to delocalize the excited electron and hole at two different ligands than on the same 2-pyridylpyrrolide ligand. A similar behaviour was observed previously in oxidative and reductive spectroelectro-chemistry studies.[1]

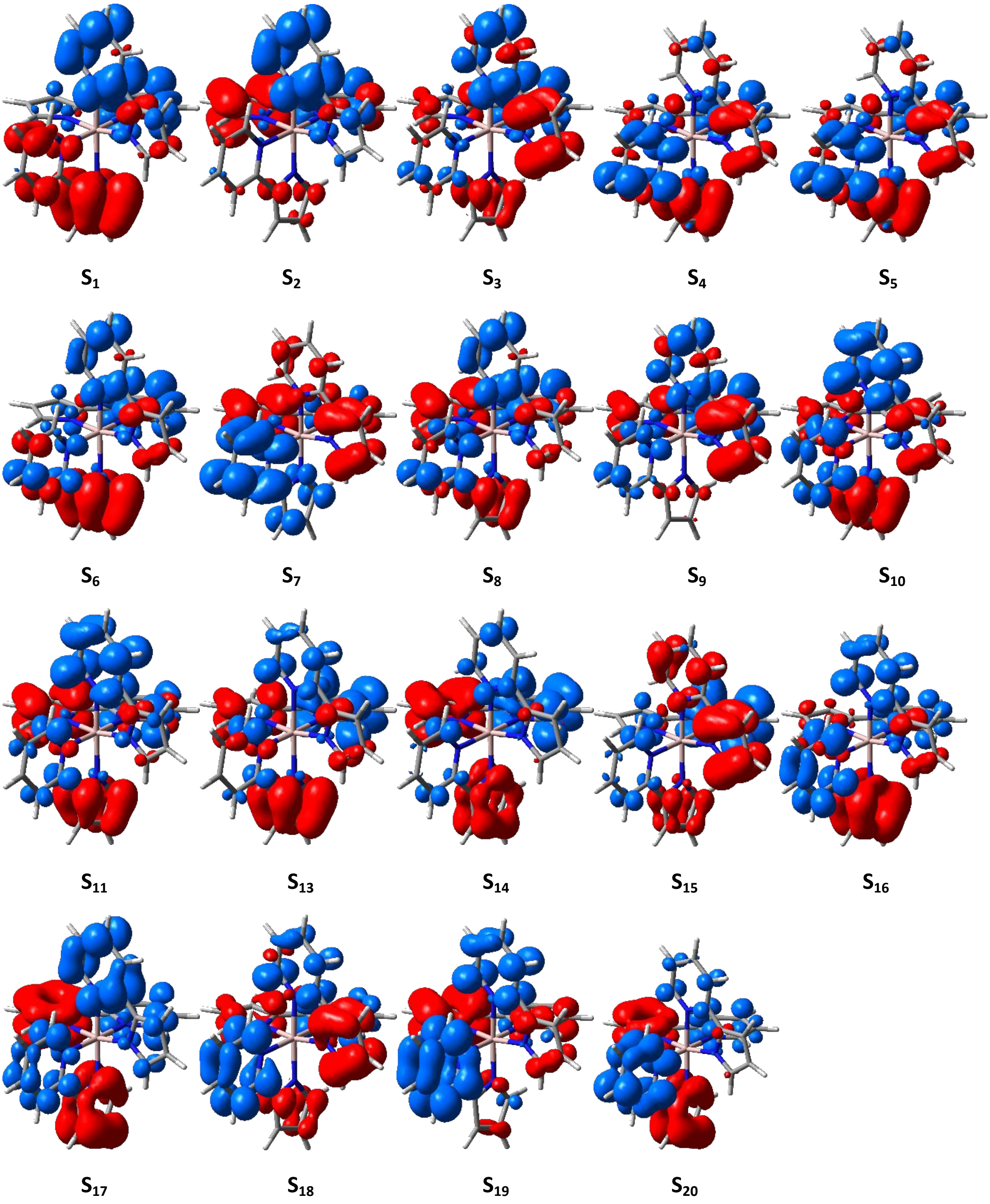

Figure S10. Electronic difference density plots of **Al1**. Electron density migrating from red to blue during the $S_0 \rightarrow S_n$ transition (densities are show using an isovalue of 0.001).

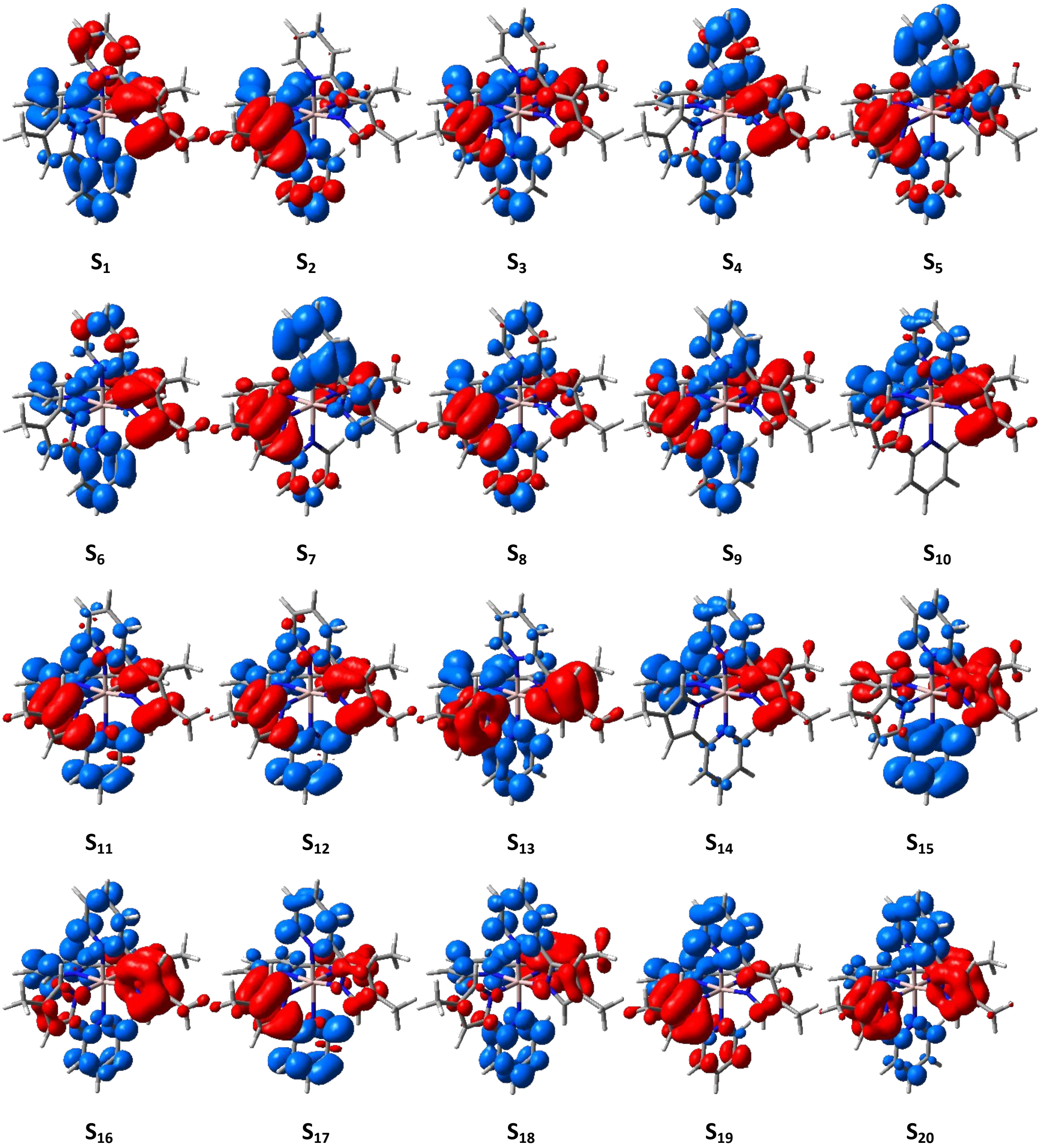

Figure S11. Electronic difference density plots of **Al2**. Electron density migrating from red to blue during the $S_0 \rightarrow S_n$ transition (densities are show using an isovalue of 0.001).

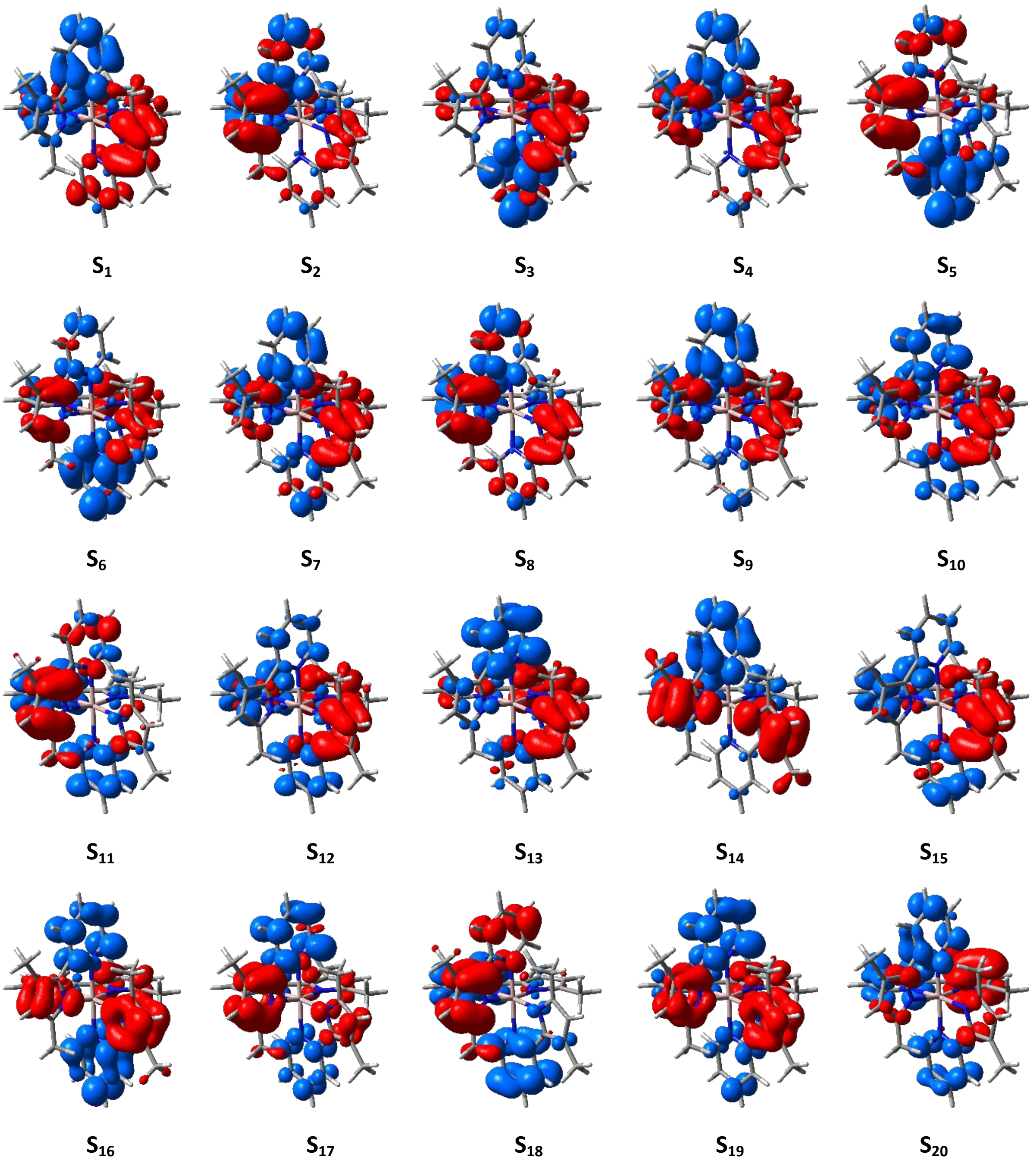

Figure S12. Electronic difference density plots of **Al3**. Electron density migrating from red to blue during the $S_0 \rightarrow S_n$ transition (densities are show using an isovalue of 0.001).

Table S5. Spin-orbit coupling elements ($\langle T_m \mid \hat{H}_{SOC} \mid S_n \rangle$ in cm^{-1}) between prominent excited singlet and triplet states of **Al1** (in chloroform). Excitation energies (*E* in eV) are given for all singlet and triplet excitations; oscillator strengths (*f*) are provided for singlet-singlet excitations only. The provided triplet states are selected to match the energy level of the highest considered singlet state (i.e. S_{10}). All results were obtained by TD-B3LYP as implemented in Orca 5.0.3.

	S_n<br>f<br>ΔE_n / eV	S_1<br>0.0620<br>3.22	S_2<br>0.0723<br>3.29	S_3<br>0.0342<br>3.34	S_4<br>0.1148<br>3.37	S_5<br>0.0711<br>3.45	S_9<br>0.1662<br>3.63	S_{10}<br>0.0972<br>3.86
T_m	ΔE_m / eV							
T_1	2.58	0.6	0.1	1.0	0.9	0.9	0.6	0.1
T_2	2.61	0.9	0.5	0.6	0.3	0.3	0.3	0.2
T_3	2.63	0.3	1.2	0.6	0.2	0.4	0.7	0.1
T_4	3.12	0.2	0.1	0.1	0.1	0.2	0.2	0.1
T_5	3.14	0.1	0.1	0.1	0.3	0.1	0.4	0.1
T_6	3.17	0.1	0.2	0.1	0.1	0.2	0.5	0.1
T_7	3.27	0.3	0.1	0.2	1.0	0.9	0.4	0.4
T_8	3.38	0.8	1.1	0.2	0.3	0.5	0.8	0.1
T_9	3.43	1.0	0.4	1.2	0.5	0.7	0.5	0.3
T_{10}	3.45	0.9	0.5	0.3	0.5	0.2	0.7	0.3
T_{11}	3.51	0.6	0.9	0.7	0.4	0.6	0.7	0.1
T_{12}	3.52	0.6	0.6	1.0	0.7	0.4	0.2	0.1
T_{13}	3.66	0.1	0.0	0.1	0.1	0.1	0.0	0.0
T_{14}	3.69	0.3	0.0	0.2	0.1	0.1	0.1	0.0
T_{15}	3.69	0.4	0.4	0.3	0.2	0.1	0.1	0.2
T_{16}	3.90	0.1	0.3	0.1	0.0	0.2	0.1	0.1

Table S6. Spin-orbit coupling elements ($\langle T_m | \hat{H}_{SOC} | S_n \rangle$ in cm^{-1}) between prominent excited singlet and triplet states of **Al2** (in chloroform). Excitation energies (*E* in eV) are given for all singlet and triplet excitations; oscillator strengths (*f*) are provided for singlet-singlet excitations only. The provided triplet states are selected to match the energy level of the highest considered singlet state (i.e. S_{10}). All results were obtained by TD-B3LYP as implemented in Orca 5.0.3.

	S_n<br>f<br>ΔE_n / eV	S_1<br>0.0601<br>3.13	S_2<br>0.0784<br>3.19	S_3<br>0.0334<br>3.24	S_4<br>0.1203<br>3.27	S_5<br>0.0717<br>3.35	S_9<br>0.1608<br>3.53	S_{10}<br>0.1019<br>3.76
T_m	ΔE_m / eV							
T_1	2.52	0.7	0.0	0.9	1.0	0.9	0.6	0.1
T_2	2.54	0.9	0.4	0.7	0.3	0.3	0.3	0.2
T_3	2.57	0.3	1.2	0.5	0.2	0.4	0.7	0.1
T_4	3.07	0.2	0.1	0.1	0.2	0.2	0.2	0.1
T_5	3.08	0.1	0.1	0.1	0.4	0.2	0.4	0.1
T_6	3.11	0.1	0.1	0.1	0.1	0.3	0.5	0.0
T_7	3.17	0.3	0.1	0.2	0.9	0.8	0.5	0.4
T_8	3.29	0.6	1.1	0.2	0.2	0.5	0.7	0.1
T_9	3.33	1.2	0.4	1.1	0.4	0.8	0.6	0.2
T_{10}	3.35	0.8	0.5	0.4	0.5	0.2	0.6	0.3
T_{11}	3.40	0.4	0.8	0.5	0.3	0.5	0.6	0.0
T_{12}	3.42	0.6	0.5	1.1	0.6	0.4	0.3	0.1
T_{13}	3.45	0.2	0.3	0.4	0.3	0.1	0.1	0.1
T_{14}	3.47	0.3	0.6	0.3	0.2	0.3	0.1	0.1
T_{15}	3.47	0.6	0.1	0.2	0.2	0.1	0.2	0.1
T_{16}	3.80	0.1	0.3	0.1	0.0	0.2	0.1	0.1

Table S7. Spin-orbit coupling elements ($\langle T_m \mid \hat{H}_{SOC} \mid S_n \rangle$ in cm^{-1}) between prominent excited singlet and triplet states of **Al3** (in chloroform). Excitation energies (E in eV) are given for all singlet and triplet excitations; oscillator strengths (f) are provided for singlet-singlet excitations only. The provided triplet states are selected to match the energy level of the highest considered singlet state (i.e. S_{17}). All results were obtained by TD-B3LYP as implemented in Orca 5.0.3.

S_n	S_1	S_2	S_3	S_5	S_7	S_8	S_9	S_{10}	S_{11}	S_{12}	S_{13}	S_{15}	S_{16}	S_{17}
f	0.0672	0.0864	0.0520	0.0457	0.0801	0.0750	0.1000	0.1688	0.0897	0.1026	0.1156	0.1105	0.0977	0.1708
ΔE_n / eV	2.98	3.04	3.09	3.18	3.30	3.36	3.44	3.64	3.67	3.76	3.79	3.85	3.89	3.90
**T_m** ΔE_m / eV														
**T_1** 2.38	0.8	0.5	1.0	0.6	1.0	0.4	0.5	0.2	0.1	0.1	0.3	0.3	0.2	0.1
**T_2** 2.40	0.7	0.5	0.5	0.5	0.8	0.6	0.3	0.2	0.1	0.1	0.2	0.2	0.2	0.2
**T_3** 2.42	0.3	0.9	0.4	0.4	0.2	0.8	0.7	0.1	0.3	0.1	0.2	0.1	0.1	0.1
**T_4** 2.94	0.3	0.1	0.3	0.3	0.7	0.3	0.4	0.2	0.1	0.0	0.1	0.3	0.1	0.1
**T_5** 2.98	0.2	0.1	0.1	0.3	0.3	0.5	0.4	0.1	0.1	0.1	0.0	0.1	0.1	0.1
**T_6** 3.00	0.1	0.2	0.2	0.4	0.2	0.8	0.3	0.1	0.1	0.1	0.1	0.1	0.1	0.1
**T_7** 3.05	0.5	0.1	0.6	0.5	0.7	0.9	0.9	0.4	0.2	0.4	0.2	0.2	0.2	0.0
**T_8** 3.15	0.4	1.1	0.2	0.5	0.5	0.5	0.4	0.2	0.3	0.1	0.1	0.2	0.3	0.1
**T_9** 3.20	0.7	0.6	0.4	0.4	0.2	0.4	0.8	0.3	0.1	0.3	0.1	0.1	0.1	0.1
**T_{10}** 3.22	1.3	0.1	0.4	0.3	0.6	0.2	0.9	0.4	0.1	0.2	0.1	0.3	0.2	0.1
**T_{11}** 3.28	0.1	1.1	0.2	0.9	0.0	0.4	0.7	0.1	0.3	0.2	0.2	0.1	0.1	0.0
**T_{12}** 3.36	0.7	0.6	0.5	0.3	0.3	0.3	0.2	0.1	0.1	0.1	0.2	0.1	0.1	0.1
**T_{13}** 3.47	0.1	0.1	0.1	0.1	0.1	0.1	0.1	0.0	0.0	0.0	0.1	0.2	0.1	0.4
**T_{14}** 3.50	0.1	0.1	0.1	0.2	0.2	0.1	0.2	0.1	0.1	0.1	0.1	0.2	0.2	0.8
**T_{15}** 3.52	0.4	0.4	0.2	0.2	0.1	0.2	0.2	0.1	0.2	0.2	0.1	0.1	0.1	0.3
**T_{16}** 3.70	0.1	0.3	0.0	0.3	0.1	0.1	0.1	0.2	0.0	0.2	0.1	0.1	0.0	0.1
**T_{17}** 3.73	0.2	0.1	0.4	0.1	0.2	0.1	0.1	0.1	0.1	0.1	0.1	0.1	0.2	0.1
**T_{18}** 3.79	0.3	0.1	0.2	0.1	0.1	0.1	0.1	0.1	0.2	0.1	0.1	0.1	0.1	0.1
**T_{19}** 3.84	0.2	0.3	0.1	0.2	0.1	0.2	0.1	0.1	0.0	0.1	0.1	0.1	0.1	0.1
**T_{20}** 3.90	0.1	0.1	0.2	0.2	0.2	0.2	0.2	0.0	0.0	0.1	0.1	0.1	0.2	0.1

Table S8. Spin densities of the optimized (unrestricted DFT; densities are show using an isovalue of 0.001) triplet ground states (T_1) of **Al1**, **Al2** and **Al3**. Relative T_1 energies are provided with respect to the S_0 in the optimized S_0 equilibrium structure, respectively.

**Al1**	**Al2**	**Al3**
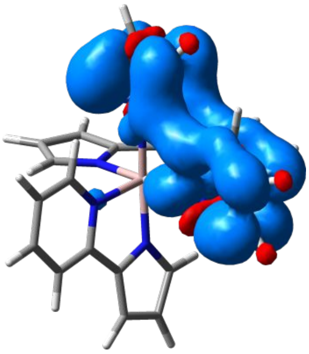	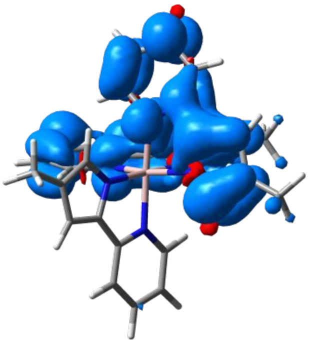	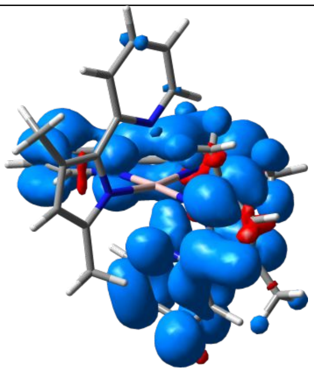
^{3}ILCT (2.47 eV)	^{3}LLCT (2.68 eV)	^{3}LLCT (2.52 eV)

5 Molecular Structures and Purity of the Reference Compounds

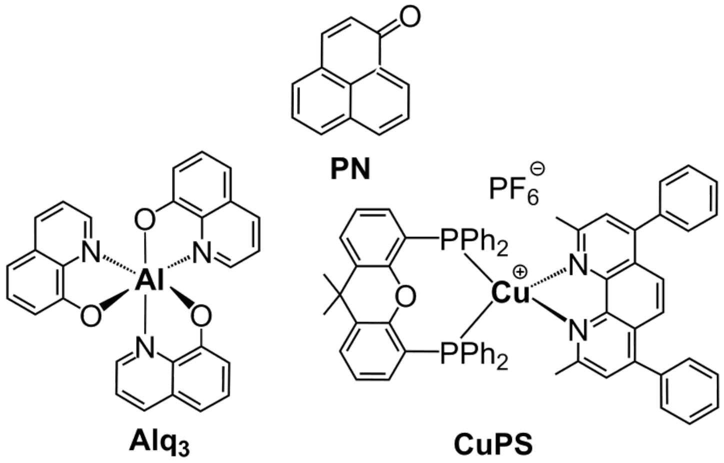

Figure S13. Molecular structures of perinaphthenone (**PN**), tris(8-hydroxyquinolinato)aluminium (**Alq$_3$**) and [Cu(bcp)(xant)]PF_6 (**CuPS**, with bcp = bathocuproine and xant = xantphos).

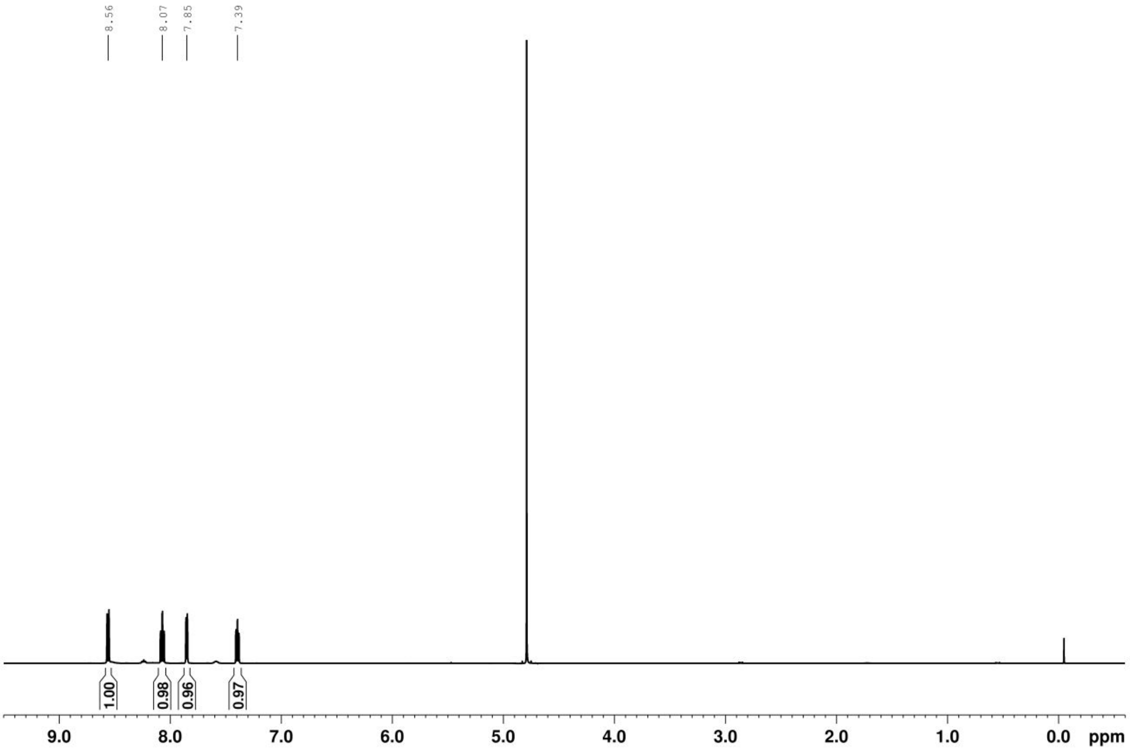

Figure S14. 1H-NMR of $(Ru(bpy)_3)Cl_2$*6 H_2O (**RuPS**, bpy = 2,2'-bipyridine) in D_2O (500 MHz).

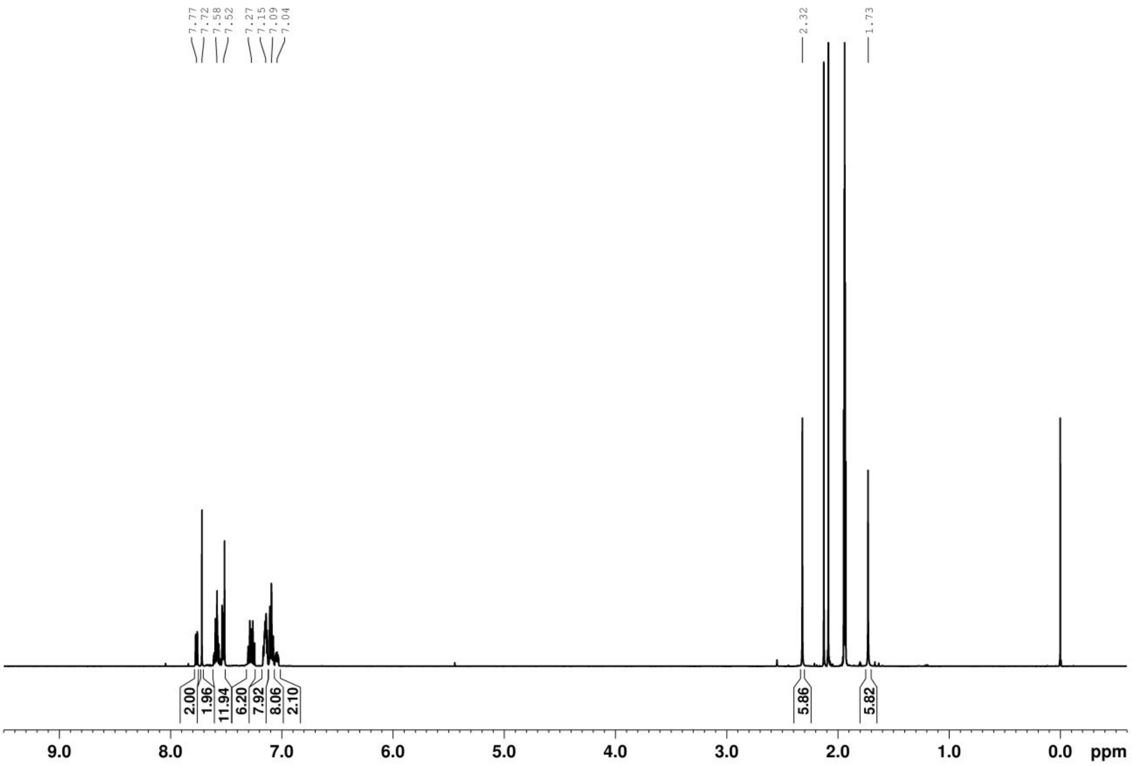

Figure S15. ^{1}H-NMR of [Cu(bcp)(xant)]PF_6 (**CuPS**) in CD_3CN (500 MHz).

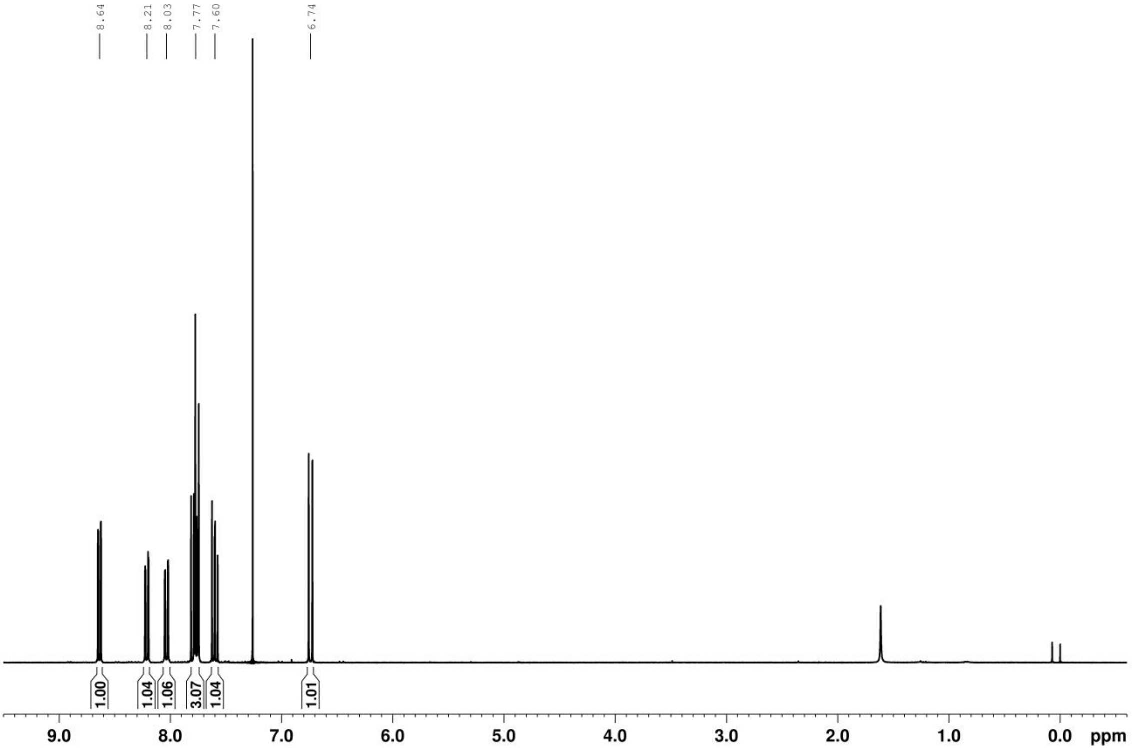

Figure S16. ^{1}H-NMR of perinaphthenone (**PN**) in $CDCl_3$ (300 MHz).

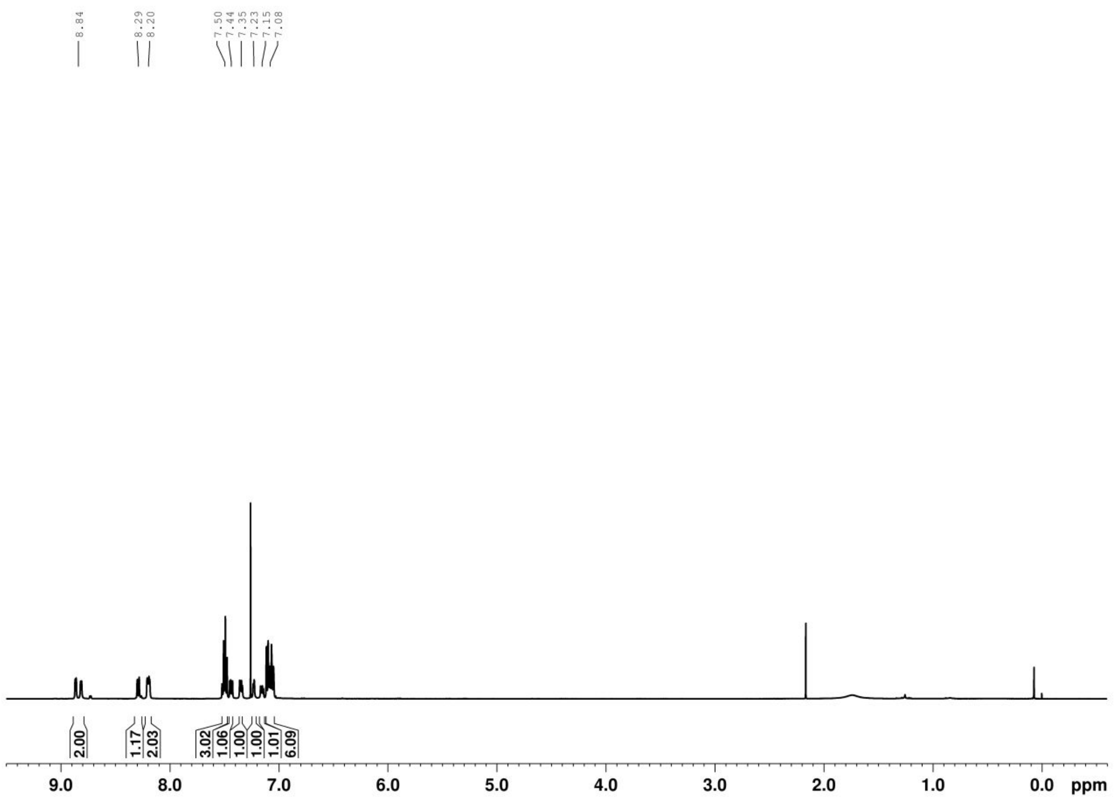

Figure S17. ^{1}H-NMR of tris(8-hydroxyquinolinato)aluminium (**Alq$_3$**) in $CDCl_3$ (500MHz).

All NMR spectra underline illustrate the high purity of the used reference compounds.[2-5] **RuPS**, **PN** and **Alq$_3$** were commercially purchased (Sigma-Aldrich) and used as received.

6 Emission Properties of Al1-3 and Alq$_3$

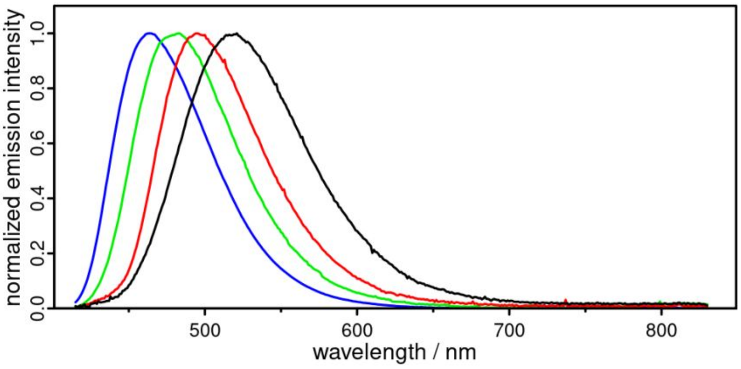

Figure S18. Normalized emission intensities of **Al1** (blue, λ_{exc} = 369 nm), **Al2** (green, λ_{exc} = 379 nm) and **Al3** (red, λ_{exc} = 400 nm) and **Alq$_3$** (black, λ_{exc} = 405 nm) with an OD_{exc} = 0.1.

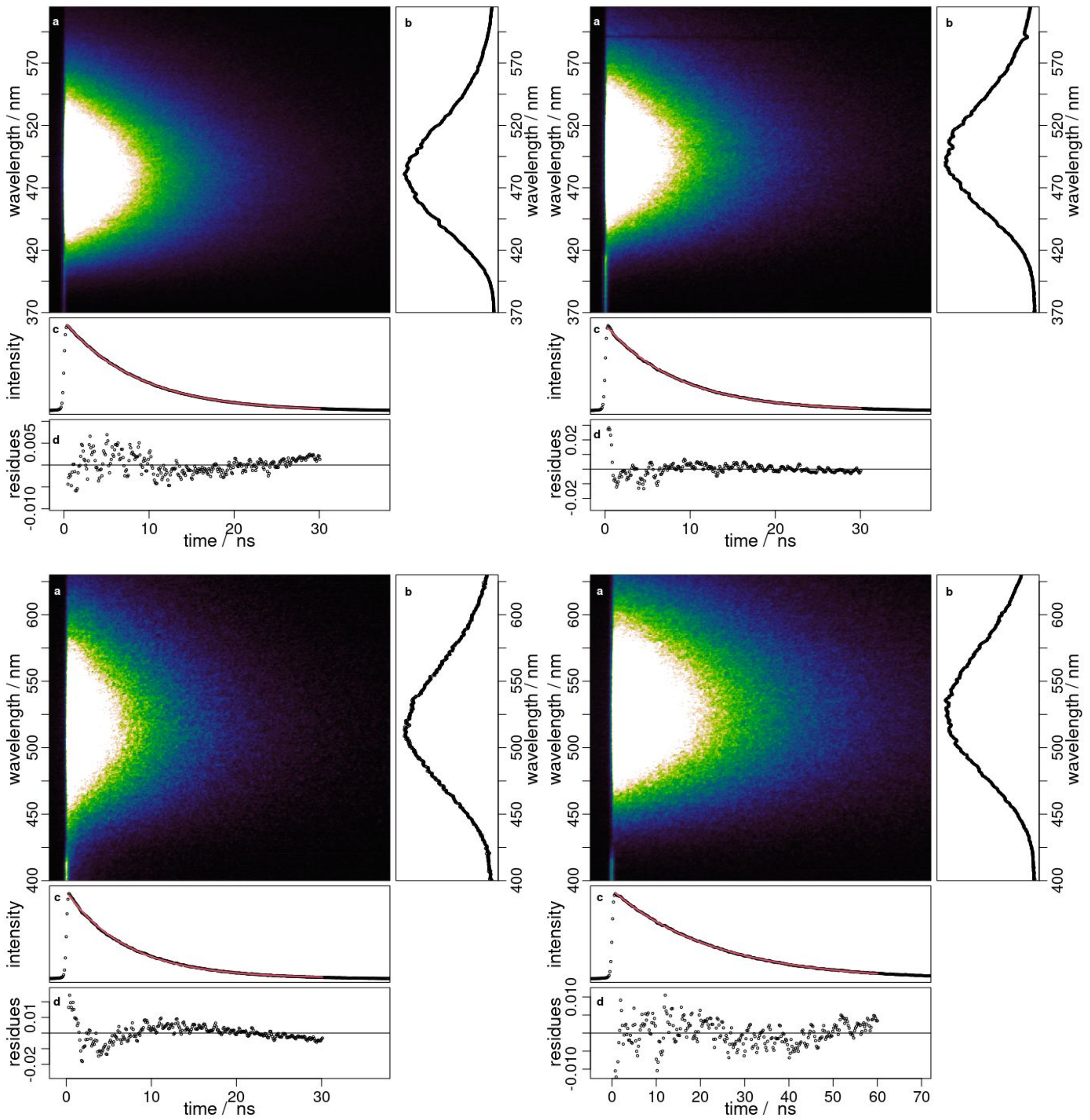

Figure S19. Streak-camera measurements of **Al1** (top left), **Al2** (top right), **Al3** (bottom left) and **Alq$_3$** (bottom right). The measurement plots show (a) the streak camera signal, (b) the emission spectra, (c) the emission decay with corresponding emission lifetime and (d) the residuals of the decay fit.

Table S9. Emission lifetimes of **Al1**, **Al2**, **Al3** in deaerated chloroform and **Alq$_3$** in deaerated acetonitrile.

Al(III)PS	τ_{em} /ns
**Al1**	8.7
**Al2**	8.6
**Al3**	7.2
**Alq$_3$**	23.3

7 Photooxidation of 2,5-Diphenylfuran

The following experiment describe the oxidation of 2,5-diphenylfuran (DPF) by singlet oxygen, which was generated by a photosensitizer (PS) upon irradiation. The used PS is mentioned in the caption of respective spectra. The experiments were prepared in a way that there was a 1:10 ratio PS:DPF and after each hour, the same (initial) amount of DPF was added again to maintain an excess of DPF. For exact concentrations, check the experimental details (*vide supra*).

The marginal rise in the *cis*-1,2-dibenzoylethylene (DBE) signal at 260 nm following the addition of DPF can be attributed to the weak absorbance of DPF at 260 nm. Nevertheless, in comparison to the DBE, DPF has a lower absorptivity, leading to a noticeable increase in signal intensity upon conversion.

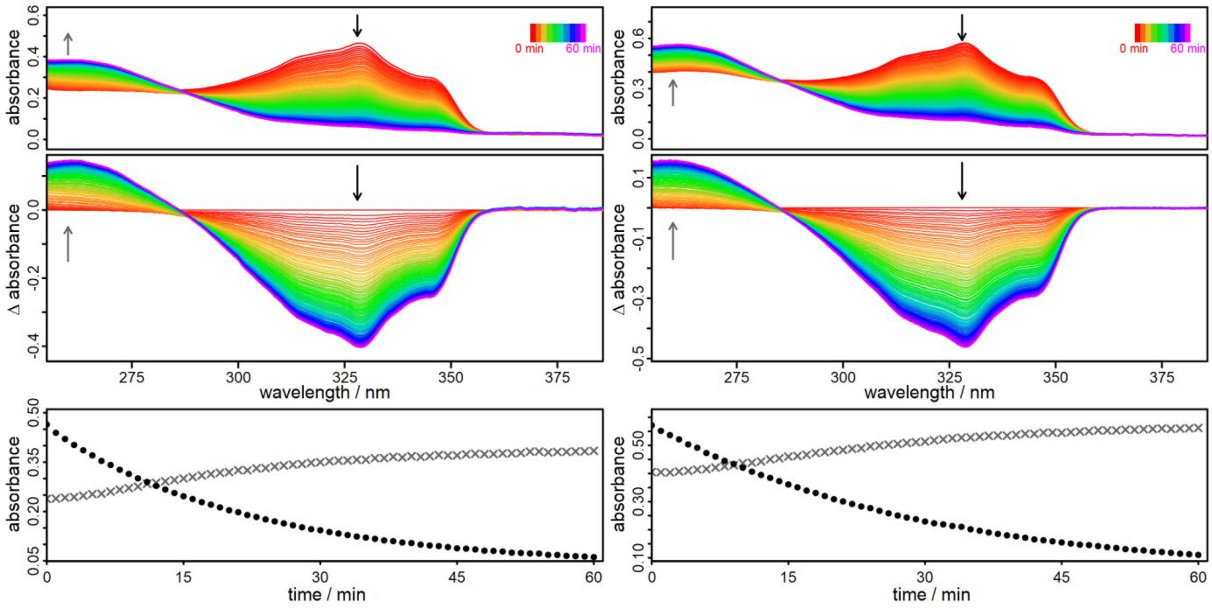

Figure S20. Subsequent DPF catalysis measurements of **Al1** after one (left) and two (right) DPF additions. Each graph contains *in situ* absorption spectra (top), difference absorption spectra (middle) and time dependent absorbance values (bottom) for the depletion of DPF (λ = 328 nm, black dots) and DBE generation (λ = 260 nm, grey crosses). Irradiation time after addition of DPF was 1 h and the irradiated solutions were not removed from the cuvette. A 150 W Xe-lamp and a 400 nm long-pass filter was used.

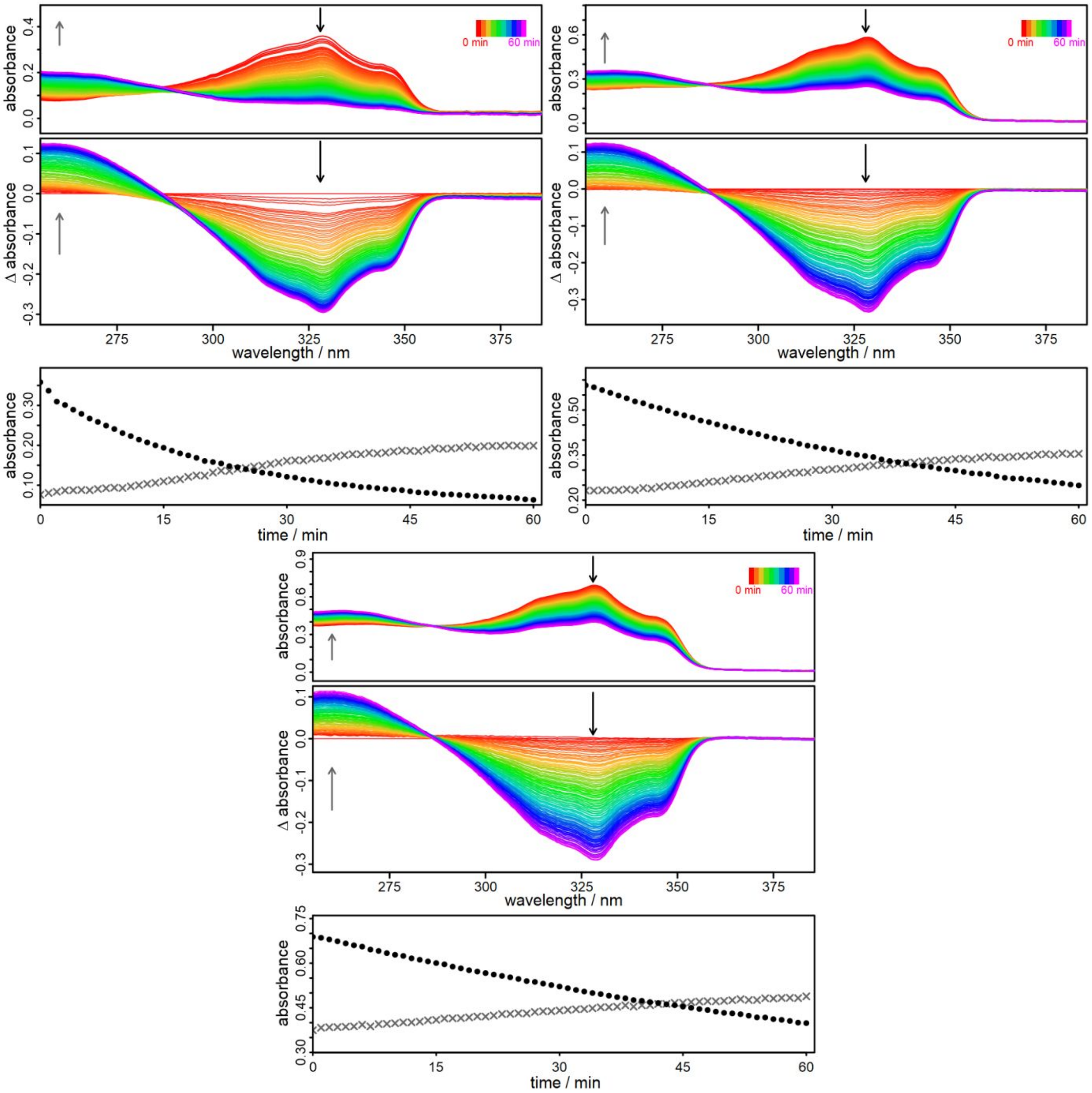

Figure S21. DPF catalysis measurements of **Al2** for the first cycle (top left), after one addition of DPF (second cycle, top right) and after a second addition of DPF (bottom). Irradiation time after addition of DPF was 1 h and the irradiated solutions were not removed from the cuvette. Each graph contains *in situ* absorption spectra (top), difference absorption spectra (middle) and time dependent absorbance values (bottom) for the depletion of DPF (λ = 328 nm, black dots) and DBE generation (λ = 260 nm, grey crosses). A 150 W Xe-lamp and a 400 nm long-pass filter was used.

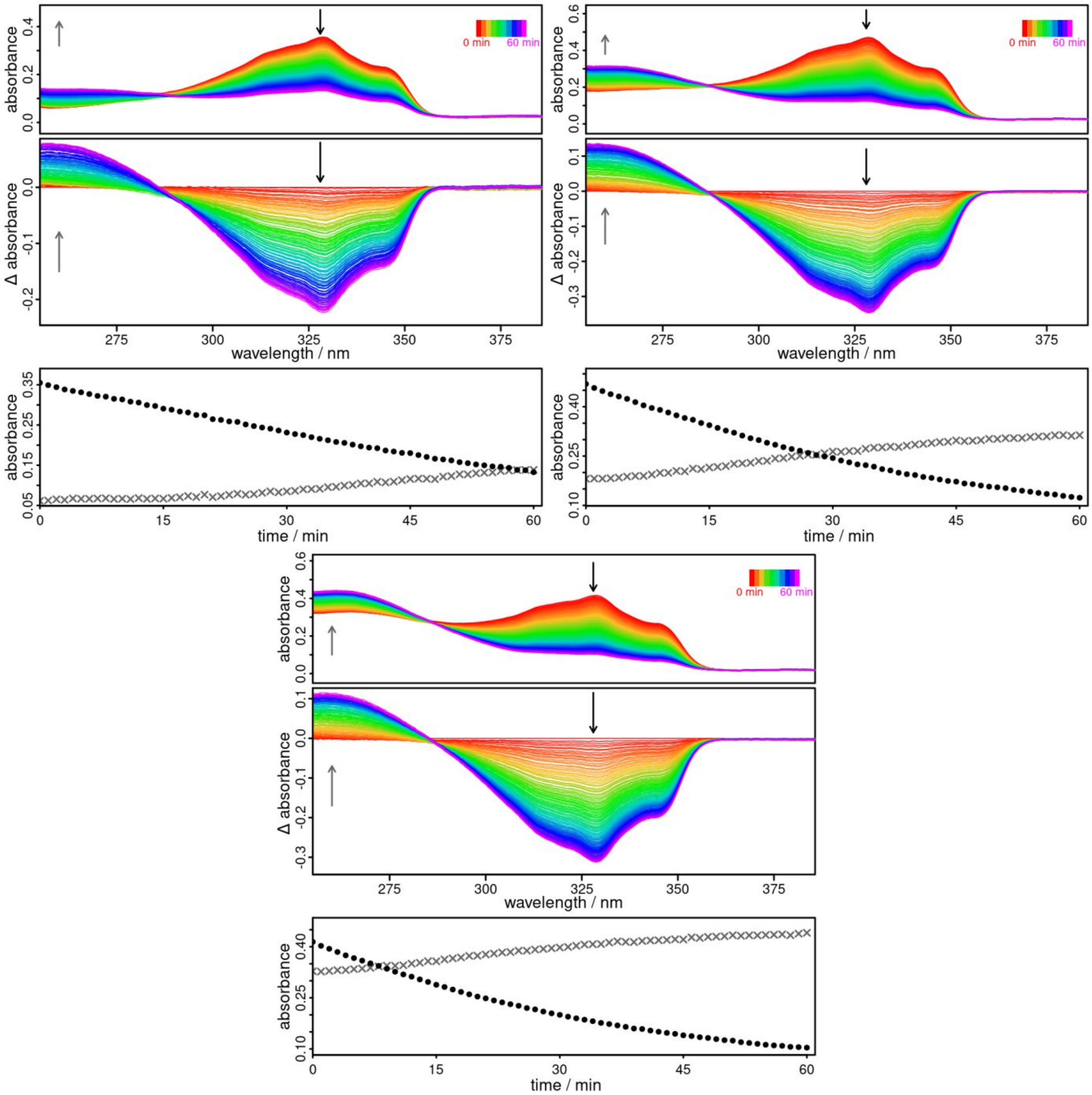

Figure S22. DPF catalysis measurements of **Al3** for the first cycle (top left), after one addition of DPF (second cycle, top right) and after a second addition of DPF (bottom). Irradiation time after addition of DPF was 1 h and the irradiated solutions were not removed from the cuvette. Each graph contains *in situ* absorption spectra (top), difference absorption spectra (middle) and time dependent absorbance values (bottom) for the depletion of DPF (λ = 328 nm, black dots) and DBE generation (λ = 260 nm, grey crosses). A 150 W Xe-lamp and a 400 nm long-pass filter was used.

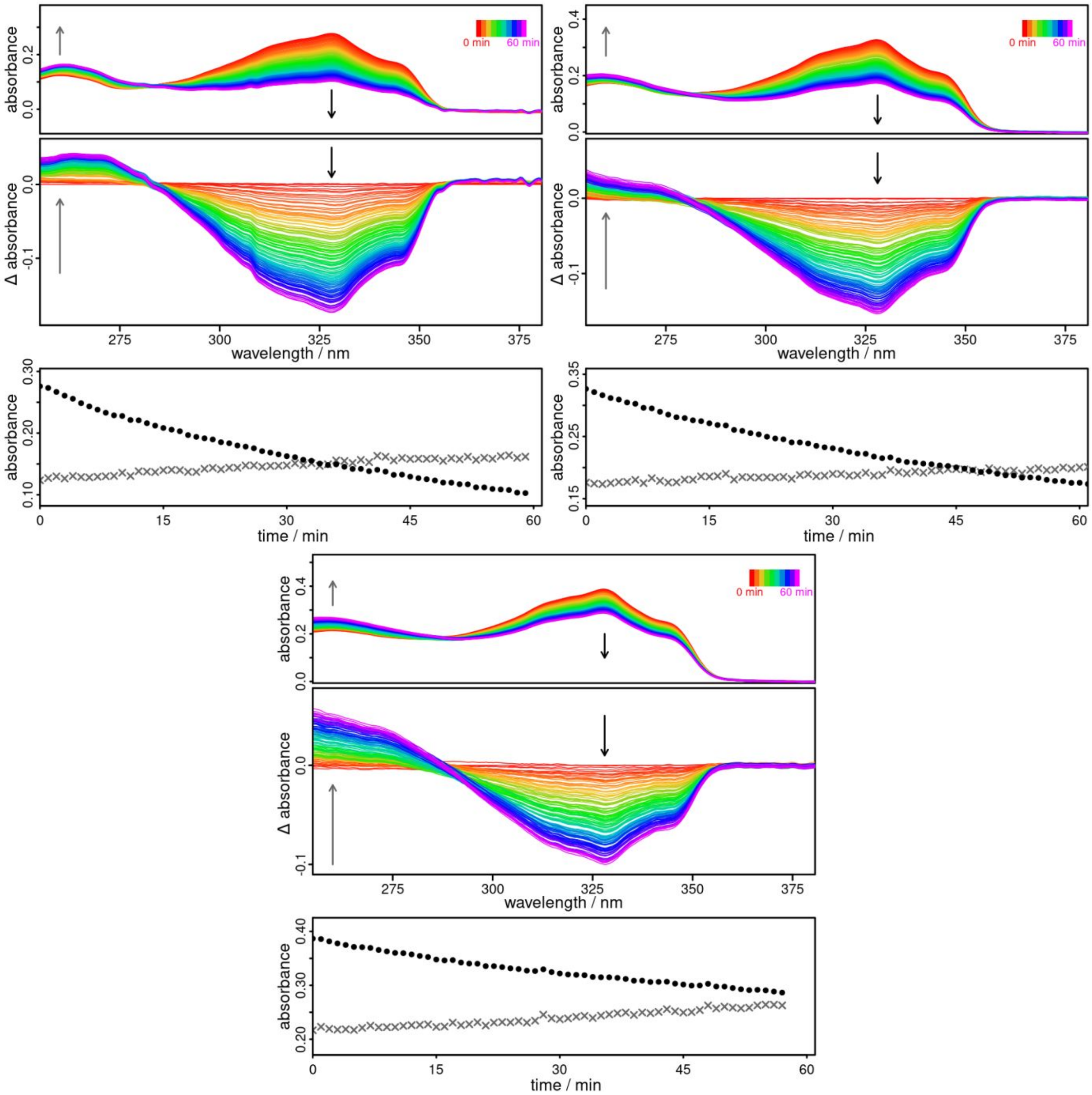

Figure S23. DPF catalysis measurements of **Alq$_3$** for the first cycle (top left), after one addition of DPF (second cycle, top right) and after a second addition of DPF (bottom). Irradiation time after addition of DPF was 1 h and the irradiated solutions were not removed from the cuvette. The graph contains *in situ* absorption spectra (top), difference absorption spectra (middle) and time dependent absorbance values (bottom) for the depletion of DPF (λ = 328 nm, black dots) and DBE generation (λ = 260 nm, grey crosses). A decrease of DPF conversion rates is observable after only 2 additions. A 150 W Xe-lamp and a 400 nm long-pass filter was used.

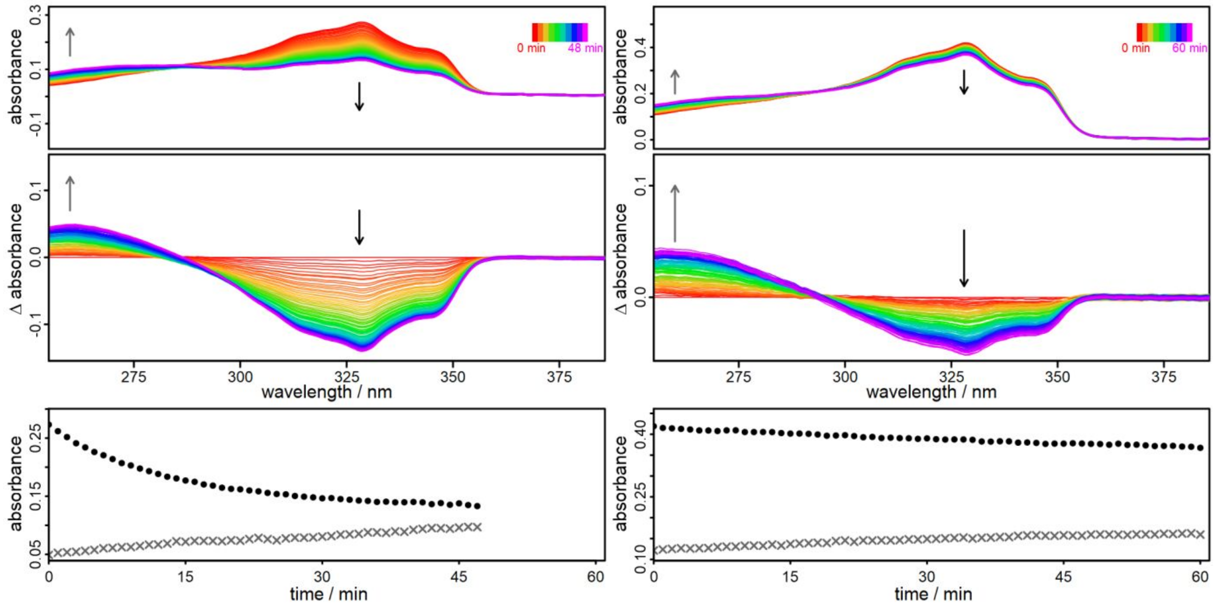

Figure S24. DPF catalysis measurements of **CuPS** for the first cycle (left) and after one addition of DPF (second cycle, right). Irradiation time after addition of DPF was 1 h and the irradiated solutions were not removed from the cuvette. Each graph contains *in situ* absorption spectra (top), difference absorption spectra (middle) and time dependent absorbance values (bottom) for the depletion of DPF (λ = 328 nm, black dots) and DBE generation (λ = 260 nm, grey crosses). A strong decrease of conversion was observable during the first cycle. A 150 W Xe-lamp and a 400 nm long-pass filter was used.

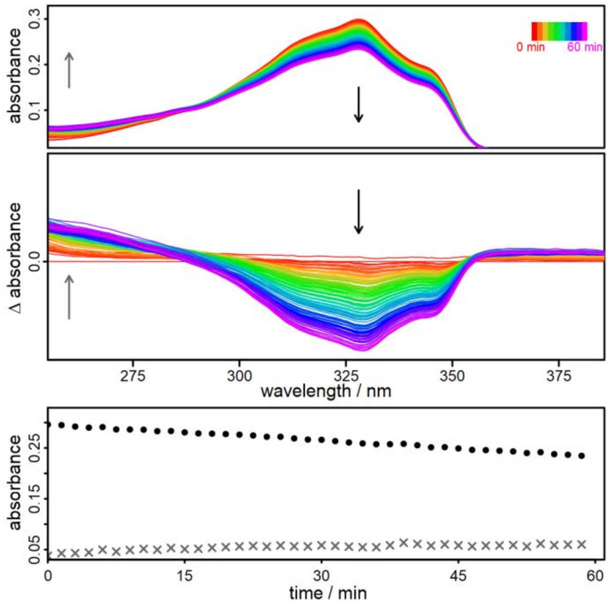

Figure S25. DPF catalysis measurements of **RuPS**. The graph contains *in situ* absorption spectra (top), difference absorption spectra (middle) and time dependent absorbance values (bottom) for the depletion of DPF (λ = 328 nm, black dots) and DBE generation (λ = 260 nm, grey crosses). A 150 W Xe-lamp and a 400 nm long-pass filter was used.

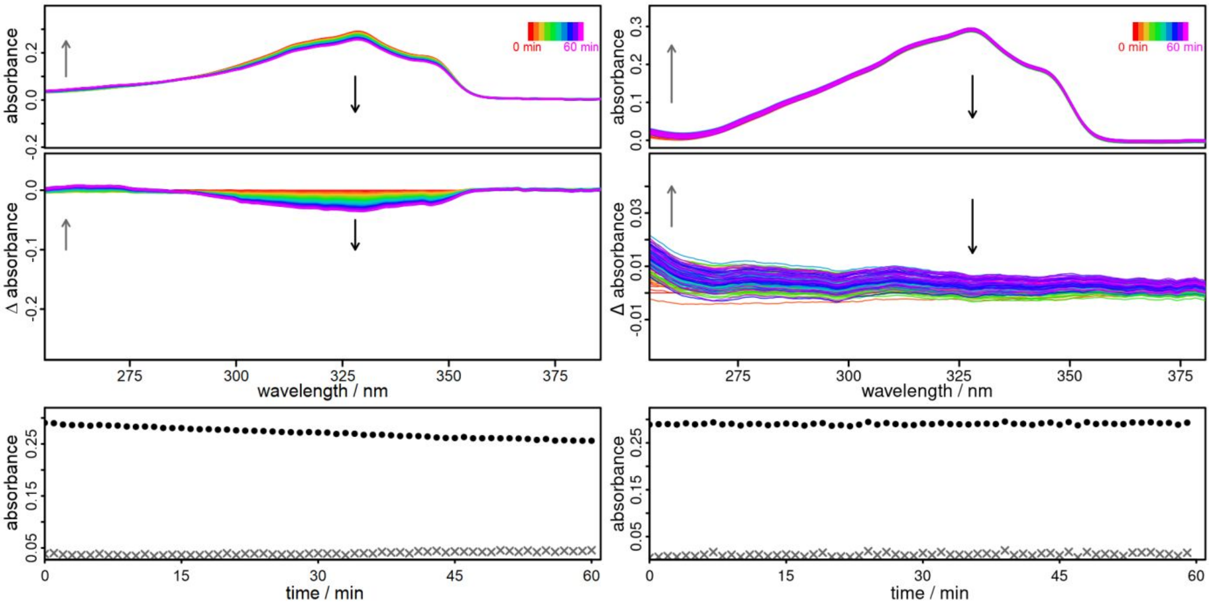

Figure S26. DPF catalysis measurements **without PS** for one cycle (top left) and with **RuPS** but **without light** (right). The graph contains *in situ* absorption spectra (top), difference absorption spectra (middle) and time dependent absorbance values (bottom) for the depletion of DPF (λ = 328 nm, black dots) and DBE generation (λ = 260 nm, grey crosses). DPF catalysis measurements without the presence of PS or light reveal no observable conversion even after 1 hour. This proves the essential role of a photosensitizer in generating $^1O_2$, which subsequently facilitates the oxidation of DPF. A 150 W Xe-lamp and a 400 nm long-pass filter was used.

8 DPF Conversion Rates for Al2, Al3, Alq$_3$, PN and CuPS

The value of the rate constants *k* is defined in equation S2 and can be interpreted as the rate constant for the DPF conversion. The rates were calculated using a

$$k = -\frac{\ln\left(\frac{c_t}{c_0}\right)}{t} = \frac{\ln\left(\frac{c_0}{c_t}\right)}{t} = \text{slope linear fit} \quad \text{(equation S2)}$$

logarithmic fit of c_0/c_t, where c_0 and c_t are the initial DPF concentration (t = 0) and the concentration of DPF after time t.

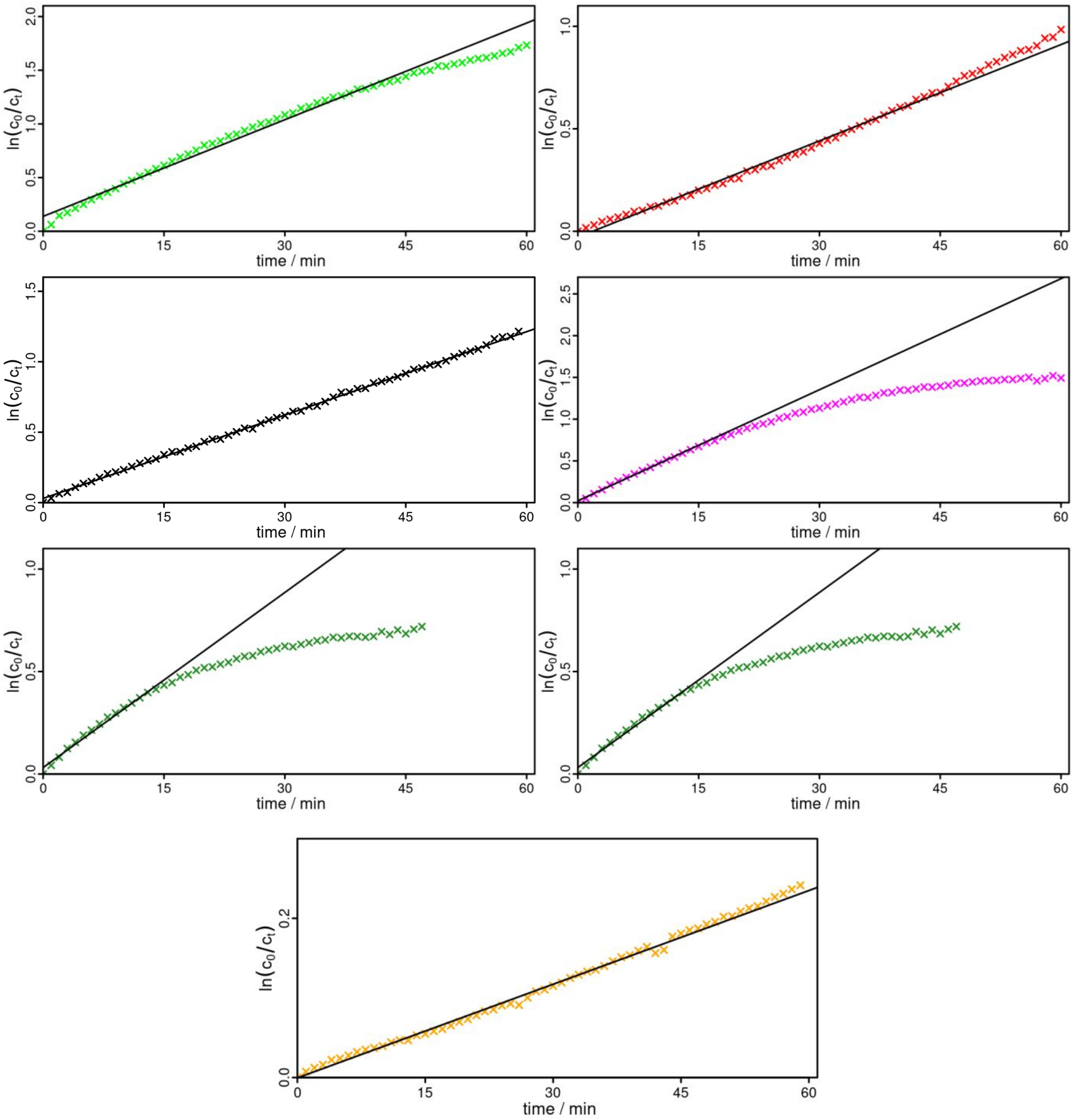

Figure S27. DPF conversion rate of the first hour (crosses) and first order fit (black line) of $\ln(c_t/c_0)$ per time. The slope of the fit is estimated to be the DPF conversion rate *k* and can be found in Table 2. Fits shown for DPF conversion experiments using **Al2** (green, top left), **Al3** (red, top right) **Alq$_3$** (black, middle left), **PN** (magenta, middle right), **CuPS** (dark green, 2nd row from bottom) and **RuPS** (orange, bottom). For **CuPS**, one fit for one hour (bottom left) and one for the first 15 min (bottom right) is given.

This approach assumes that the kinetic behavior during DPF conversion is similar for all complexes, which is the case for the first 50 min for most complexes used. The reference compounds **CuPS** as well as perinapthenone (**PN**, Figure S10), which is known to have a singlet oxygen yield close to unity, exhibits a lower photoactivity over time (Figure S19). In the case of **CuPS** and **PN**, the degradation process of the conversion happens so fast that a first order kinetic fit was only feasible for the first 15 min.

9 Isomerization of (*E*)-Stilbene

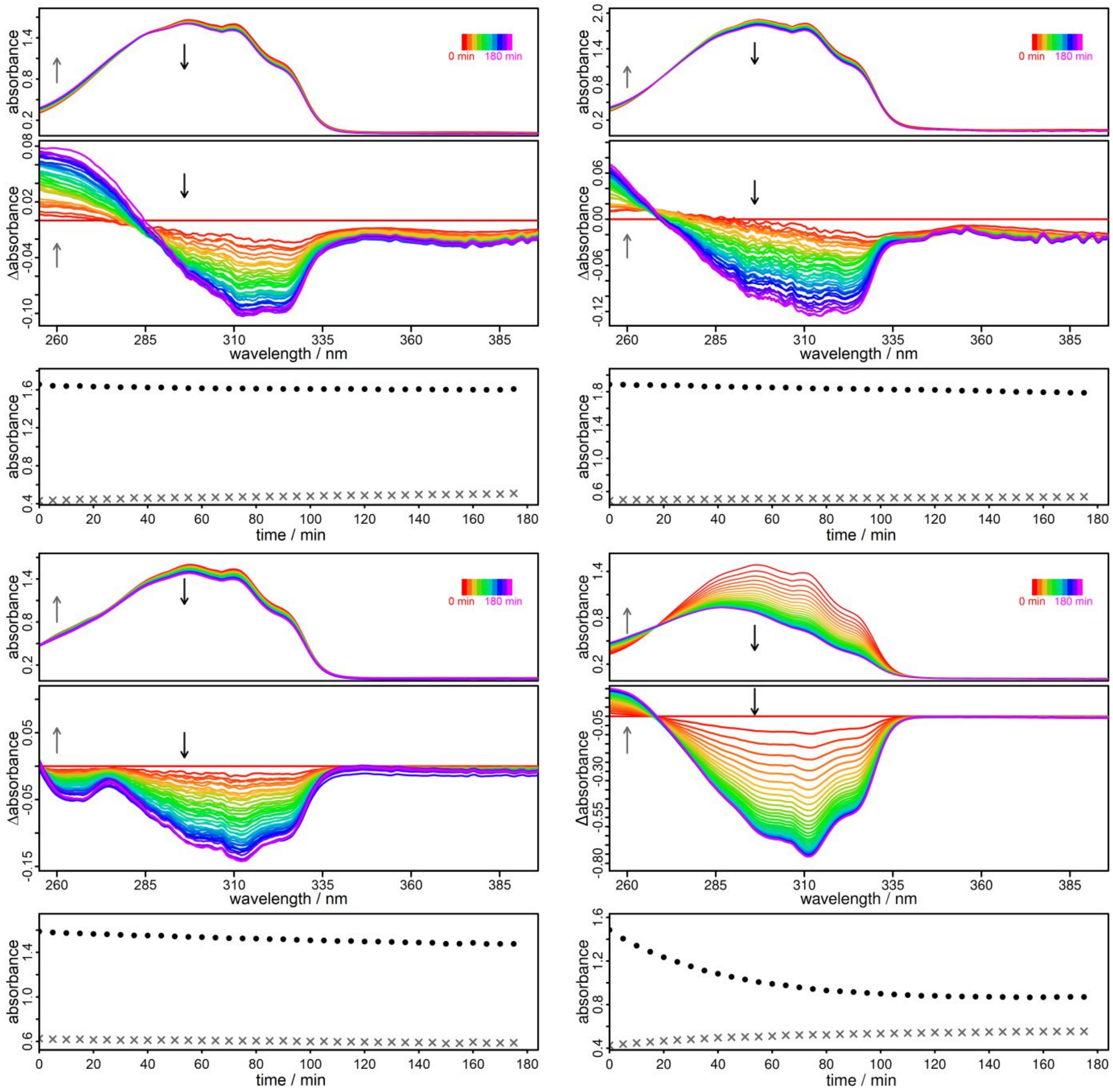

Figure S28. Photosensitized isomerization of *E*-stilbene with **Al2** (top left), **Al3** (top right), **Alq3** (bottom left), **CuPS** (bottom right) in chloroform under inert conditions. The mole ratio of photosensitizer to *E*-stilbene was 1:25. *In situ* UV/vis absorption spectra (top), the differential plot (middle) and the kinetic plots (bottom) of *E*-stilbene depletion at 296 nm (black dots) and Z-stilbene generation at 260 nm (grey crosses). A 405 nm power LED (~10.7 W) and a 400 nm long-pass filter was used.

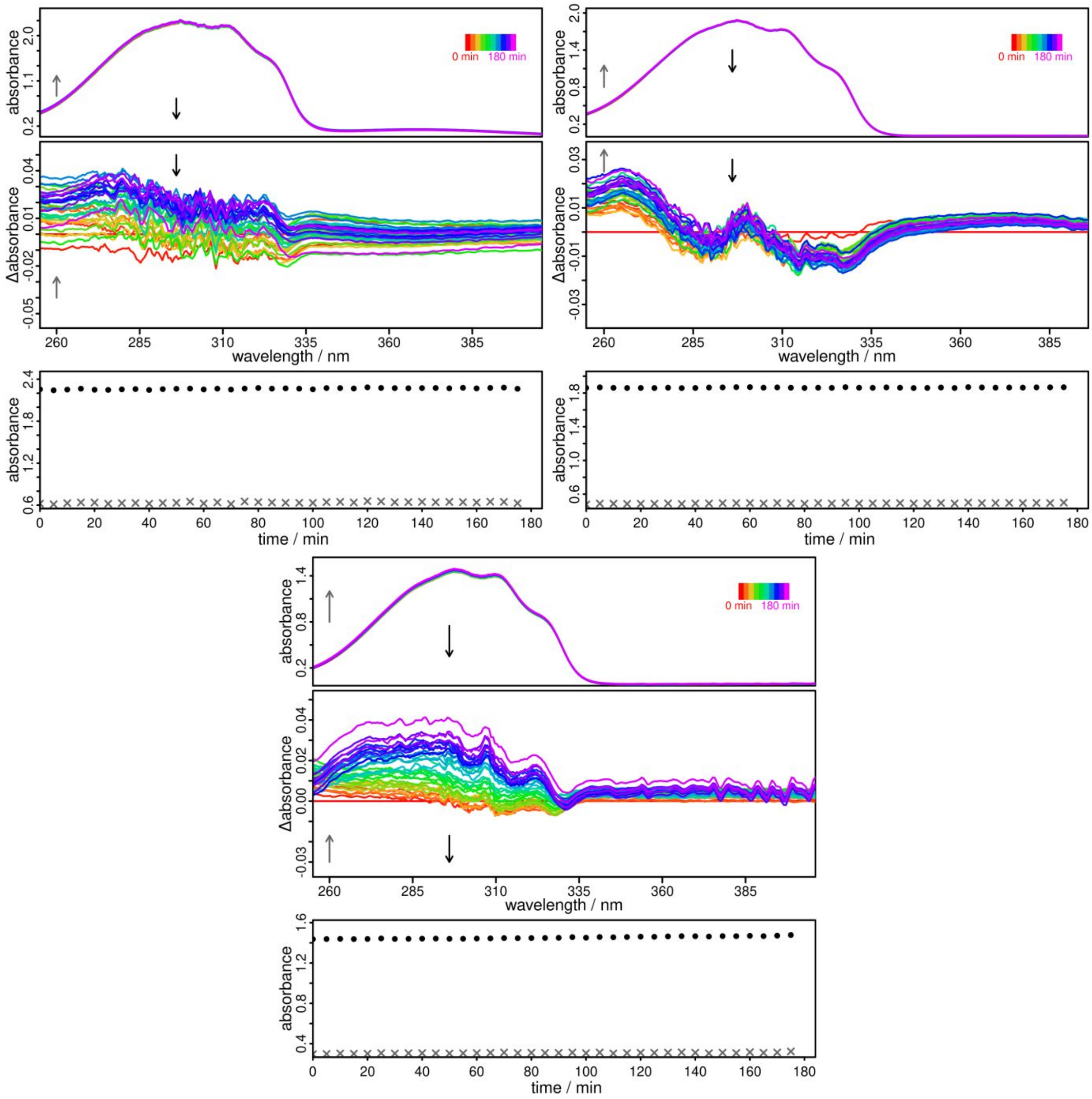

Figure S29. Photosensitized isomerization of *E*-stilbene with **Al1** but **no light** (top left), **RuPS** (top right) and **no PS** (bottom) in chloroform under inert conditions. The mole ratio of photosensitizer to *E*-stilbene was 1:25. *In situ* UV/vis absorption spectra (top), the differential plot (middle) and the kinetic plots (bottom) of *E*-stilbene depletion at 296 nm (black dots) and Z-stilbene generation at 260 nm (grey crosses). A 405 nm power LED (~10.7 W) and a 400 nm long-pass filter was used.

10 References

(1) Wang J.-W.; Ma F.; Jin T.; He P.; Luo Z.-M.; Kupfer S.; Karnahl M.; Zhao F.; Xu Z.; Jin T.; Lian T.; Huang Y.-L.; Jiang L.; Fu L.-Z.; Ouyang G.; Yi X.-Y. Homoleptic Al(III) Photosensitizers for Durable CO2 Photoreduction. *J. Am. Chem. Soc.* **2023**, 145 (1), 676–688.

(2) Chen S.; Wan Q.; Badu-Tawiah A. K. Picomole-Scale Real-Time Photoreaction Screening: Discovery of the Visible-Light-Promoted Dehydrogenation of Tetrahydroquinolines under Ambient Conditions. *Angew. Chem. Int. Ed.* **2016**, 55, 9345.

(3) Mejía E.; Luo S.-P.; Karnahl M.; Friedrich A.; Tschierlei S.; Surkus A.-E.; Junge H.; Gladiali S.; Lochbrunner S.; Beller M. A Noble-Metal-Free System for Photocatalytic Hydrogen Production from Water. *Chem. Eur. J.* **2013,** 19 (47), 15972–15978.

(4) Godard J.; Brégier F.; Arnoux P.; Myrzakhmetov B.; Champavier Y.; Frochot C.; Sol V. New Phenalenone Derivatives: Synthesis and Evaluation of Their Singlet Oxygen Quantum Yield. *ACS Omega* **2020**, 5 (43), 28264–28272.

(5) Li H.; Zhang F.; Wang Y.; Zheng D. Synthesis and characterization of tris-(8-hydroxyquinoline)aluminum. *Materials Science and Engineering B100* **2003,** 40–46.
